# Architecting Anisotropic Force‐Responsive Bidirectional High‐Contrast Three‐Color Fluorescence Switches via Effective Molecular Modulation for High‐Security Information Storage

**DOI:** 10.1002/advs.78253

**Published:** 2026-10-11

**Authors:** Yijie Zou, Zhao Chen, Shuang Li, Dian‐dian Deng, Hui‐juan Zhang, Hai‐feng He, Ya Yin, Zujin Zhao

**Affiliations:** ^1^ Jiangxi Provincial Key Laboratory of Organic Functional Molecules Institute of Organic Chemistry Jiangxi Science and Technology Normal University Nanchang People's Republic of China; ^2^ School of Chemistry and Chemical Engineering Jiangxi Science and Technology Normal University Nanchang People's Republic of China; ^3^ State Key Laboratory of Coordination Chemistry School of Chemistry and Chemical Engineering Nanjing University Nanjing People's Republic of China; ^4^ State Key Laboratory of Luminescent Materials and Devices Guangdong Provincial Key Laboratory of Luminescence from Molecular Aggregates South China University of Technology Guangzhou People's Republic of China

**Keywords:** anisotropic force, dibenzofulvene, hypsochromic and bathochromic bidirectional, information storage, molecular engineering, three‑color mechanofluorochromism

## Abstract

Although bidirectional three‐color fluorescent molecular switches triggered by anisotropic force are promising for enhancing the security of information storage, the engineering and development of such force‐responsive smart emissive materials face significant challenges, rendering them extremely rare. Here, 13 uncommon tricolored mechanofluorochromic luminogens were obtained through efficient molecular engineering strategies synergistically integrating substituent variation and positional isomeric approaches, three of which exhibited exceedingly scarce force‐induced hypsochromic and bathochromic bidirectional fluorescence responses. More specifically, employing dibenzofulvene as the core scaffold, a family of 20 fluorene‐based fluorogenic compounds was elaborately constructed by incorporating pyrene, tetraphenylethene, and three isomeric carbazole moieties at the conjugated extension sites, with phenyl and regioisomeric trifluoromethylphenyl groups respectively attached to the two flanking positions. Next, a three‐step high‐efficiency screening protocol was devised for these luminogens: first assessing mechanoresponsive fluorescence behaviors, then evaluating the three‐color mechanofluorochromic capabilities, and finally determining whether the selected force‐responsive tricolored fluorescence switches could realize bidirectional three‐color fluorescence switching after sequential grinding from gentle to vigorous. Ultimately, 3 luminogens featuring ultra‐rare bidirectional three‑color mechanofluorochromism were identified. Impressively, based on the screened force‐triggered bidirectional high‐contrast tricolor fluorescent switch **TPE‐*o*‐CF_3_**, two advanced dual‑layer information storage systems were established, which could effectively safeguard the security of the stored information.

## Graphical abstract

Synergistically integrating substituent variation and positional isomerism in molecular engineering affords 13 rare tricolored mechanofluorochromic luminogens, and 3 exceedingly scarce force‐triggered bidirectional high‐contrast three‐color fluorescence switches are screened out via a three‐step protocol. Furthermore, two high‐security dual‐layer information storage systems are established.

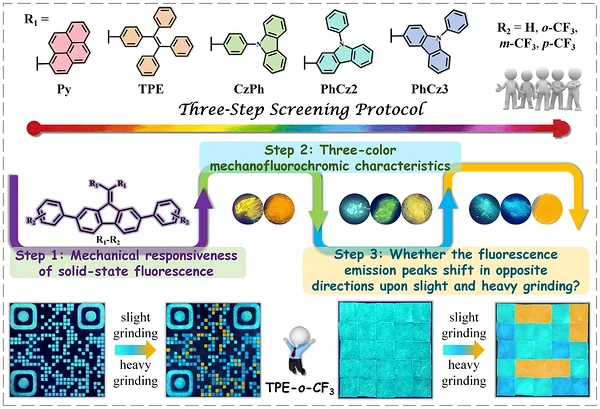

## 1 Introduction

With big data developing at an unprecedented pace and information security becoming increasingly critical, there is a soaring demand for innovative storage materials that combine large capacity with reliable security protection [1, 2, 3, 4, 5, 6]. Among the various candidates, luminescent materials have emerged as a focal point of research, not only because their photophysical characteristics are readily adjustable via molecular engineering, but also due to their innate merits, including high optical contrast and nondestructive information retrieval [7, 8, 9, 10, 11]. However, conventional emissive materials typically rely on static optical signals, which are easily replicated or deciphered, thereby compromising their effectiveness in information protection. In response to this drawback, stimuli‐responsive luminescent materials have emerged as a major research focus and have been widely deployed in information security over recent decades. Their emission characteristics are dynamically switchable upon external stimulation, facilitating advanced functionalities such as data encryption, anti‐counterfeiting, and hierarchical authentication [12, 13, 14, 15, 16]. Of the various stimuli available, anisotropic mechanical force offers a compelling advantage in terms of operational ease and direct applicability, requiring no specialized equipment. Leveraging this feature, anisotropic force‐triggered luminescent switches have gained considerable traction as promising candidates for information security in practical settings [17, 18, 19, 20, 21].

Most mechanoresponsive luminogens reported to date exhibit reversible two‐color emission switching, originating from transformations between distinct molecular conformations or packing modes [22, 23, 24, 25, 26, 27]. However, for high‐security information storage, multi‐color‐changing intelligent luminescence materials are urgently needed because they can provide richer signal states to expand encoding capacity and hinder counterfeiting. Although the last few decades have witnessed significant progress in the development of externally responsive multicolor fluorescence‐switching molecules [28, 29, 30, 31, 32, 33], a persistent bottleneck remains: the vast majority of current systems rely on combinatorial stimulation to access three or more emission states. This reliance inevitably introduces procedural intricacy and undermines their broader applicability [34, 35, 36, 37, 38, 39]. Indeed, tricolor fluorescence molecular switches driven by a single type of anisotropic force are rare; moreover, even among these few systems, most tend to exhibit only a gradual red‑shift in fluorescence emission as the grinding intensity ramps up from mild to vigorous [40, 41, 42, 43, 44, 45], and anisotropic force‐responsive bidirectional three‐color fluorescence switches are exceptionally scarce [46, 47, 48, 49]. Force‐triggered hypsochromic/bathochromic bidirectional tricolor fluorescence switches can realize three distinct fluorescence signals based on a single luminophore. From the perspective of information storage, three‐color fluorescence switching can provide a higher encoding dimensionality and richer signal combinations, thereby significantly enhancing information storage capacity. Meanwhile, since this three‐color switching relies solely on a single type of mechanical force, its operation is relatively simple, which facilitates controllable writing and reading in practical applications. Furthermore, if this extremely rare bidirectional three‐color mechanofluorochromism is applied to information storage, it will also significantly improve the security of information storage. Unfortunately, the development of smart luminogens with such intriguing phenomenon remains a formidable challenge. Given the extreme scarcity of anisotropic‑force‑triggered bidirectional tricolor fluorescence switches and the fact that their development heavily relies on serendipitous discovery, it is imperative to develop systematic molecular structural modification strategies for the rational construction of such mechanoresponsive tricolor fluorescence switches [50, 51, 52, 53, 54, 55]. In 2024, we serendipitously discovered an unprecedented luminogen featuring force‐induced bidirectional fluorescence responses [47]. This remarkable phenomenon motivated us to develop a systematic molecular engineering strategy. Herein, based on the dibenzofulvene scaffold of this reported fluorogenic molecule and through the integration of substituent diversity with regioisomeric modulation (Figure 1a), we aim to remedy the extreme scarcity of bidirectional tricolored mechanofluorochromic luminogens. A family of 20 fluorene‐based fluorogenic compounds was smoothly constructed through the molecular engineering strategy described above, and 3 luminogens with extremely rare bidirectional three‑color mechanofluorochromism were successfully developed by means of a three‑step screening method (Figure 1b). Furthermore, two advanced dual‑layer information storage systems were established to effectively secure the stored information based on one of the three screened bidirectional tricolor mechanofluorochromic switches.

**FIGURE 1:**
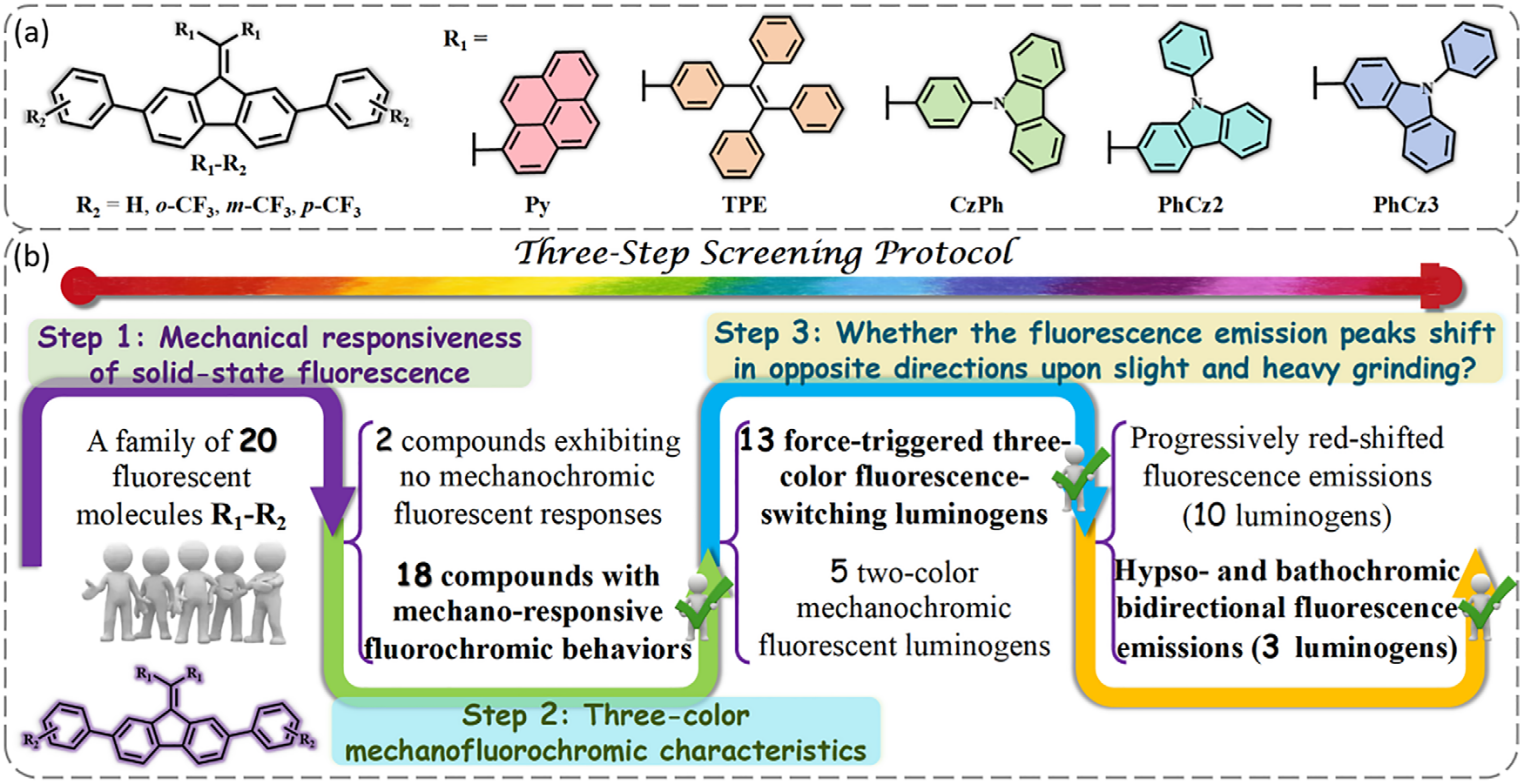
(a) Molecular structures of the 20 **R_1_‐R_2_** fluorogenic compounds engineered via structural modification. (b) Schematic representation of a three‐step screening protocol for the identification of force‐responsive hypso‐ and bathochromic bidirectional three‐color fluorescence switches.

The intermediates **1a‐1d** and **2a‐2d** were readily prepared in good yields via a two‐step sequence, and the target fluorophores **R_1_‐R_2_** were synthesized through palladium‐catalyzed Suzuki cross‐coupling (R_1_ = Py, TPE, CzPh, PhCz2, and PhCz3; R_2_ = H, *o*‐CF_3_, *m*‐CF_3_, and *p*‐CF_3_). The synthetic routes and detailed structural characterizations are provided in the Scheme S1.

## 2 Results and Discussion

### 2.1 Anisotropic Force‐Responsive Fluorescence Characteristics

In accordance with the three‐step screening protocol, our initial evaluation focused on the mechanical responsiveness of solid‑state fluorescence of 20 as‐prepared compounds bearing a dibenzofulvene skeleton. Among the 20 luminogens, 18 exhibited distinct fluorescence color changes upon mechanical stimulation, whereas **PhCz3‐*m*‐CF_3_** and **PhCz3‐*p*‐CF_3_** showed negligible mechanofluorochromic responses. As shown in Figure 2a, although mechanical force caused a bathochromic shift of the maximum emission peak (*λ*_max_) from 613 nm to 629 nm, the orange fluorescence of solid **PhCz3‐*m*‐CF_3_** showed negligible color change. The Commission Internationale de L'Eclairage (CIE) 1931 chromaticity coordinates for the solid‑state emission of pristine and ground **PhCz3‐*m*‐CF_3_** lay within the same color region, consistent with the negligible visual difference (Figure S1). To gain further insight into the fluorescence behavior of solid **PhCz3‐*m*‐CF_3_** under external force, powder X‑ray diffraction (XRD) and differential scanning calorimetry (DSC) experiments were conducted. As displayed in Figure 2e, both the pristine and ground solid samples of **PhCz3‐*m*‐CF_3_** exhibited broad diffuse scattering features in their XRD patterns, confirming that the compound remained amorphous under both conditions. Furthermore, the DSC curves (Figure 2i) revealed no discernible endothermic or exothermic peaks for solid **PhCz3‐*m*‐CF_3_** before or after grinding, suggesting the absence of mechanically induced phase transition or crystallization. Collectively, these results confirmed that mechanical grinding left the solid‑state phase of **PhCz3‐*m*‐CF_3_** unchanged, and the structural stability throughout correlated with its lack of mechanochromic fluorescence. A similar phenomenon was also observed for luminogen **PhCz3‐*p*‐CF_3_** (Figure S2). This initial screening effectively eliminated the two compounds with no fluorescence response to mechanical force, leaving the remaining 18 mechano‑responsive fluorochromic luminogens for subsequent studies. Next, the 18 force‐responsive smart luminogens were subjected to a detailed investigation to evaluate their tricolored mechanofluorochromic characteristics under sequential grinding from gentle to vigorous. Gentle grinding was performed by lightly pressing or sliding a pestle across the powder under minimal force. Of the 18 luminogens investigated, 5 emitters displayed conventional bicolor fluorescence switching after grinding, whereas the other 13 showed three distinct emission states under varying mechanical forces. For the five two‐color mechanofluorochromic luminogens, as Figure 2b shows, the pristine solid **PhCz3‐*o*‐CF_3_** emitted bright orange‑yellow fluorescence with a *λ*_max_ of 589 nm; upon grinding, the emission turned orange and red‑shifted to 620 nm (Figure S3). Notably, fuming the ground **PhCz3‐*o*‐CF_3_** sample with CH_2_Cl_2_ vapor restored its original orange‑yellow emission, confirming the reversibility of the mechanofluorochromic behavior. Powder XRD (Figure 2f) revealed that the as‑synthesized **PhCz3‐*o*‐CF_3_** exhibited sharp diffraction peaks, indicating a crystalline phase. After grinding, all crystalline diffraction peaks disappeared, indicating the occurrence of amorphization. Upon subsequent CH_2_Cl_2_ vapor exposure, the original diffraction pattern was nearly fully restored, confirming the perfect restoration of the initial crystalline state. As can be seen in Figure 2j, the ground **PhCz3‐*o*‐CF_3_** sample showed a distinct exothermic peak at 190°C in its DSC curve, which is attributed to recrystallization from the amorphous phase. The above results indicated that the two‑color fluorescence change of **PhCz3‐*o*‐CF_3_** before and after grinding originated from the mechanical‑stimulus‑induced transition from the stable crystalline state to the unstable amorphous state. The photoluminescence (PL) spectra, fluorescence photographs, and CIE 1931 chromaticity coordinates of the other four two‐color mechanofluorochromic compounds (**TPE‐H**, **TPE‐*m*‐CF_3_**, **TPE‐*p*‐CF_3_**, and **CzPh‐H**) in different solid states are presented in Figures S4–S7.

**FIGURE 2:**
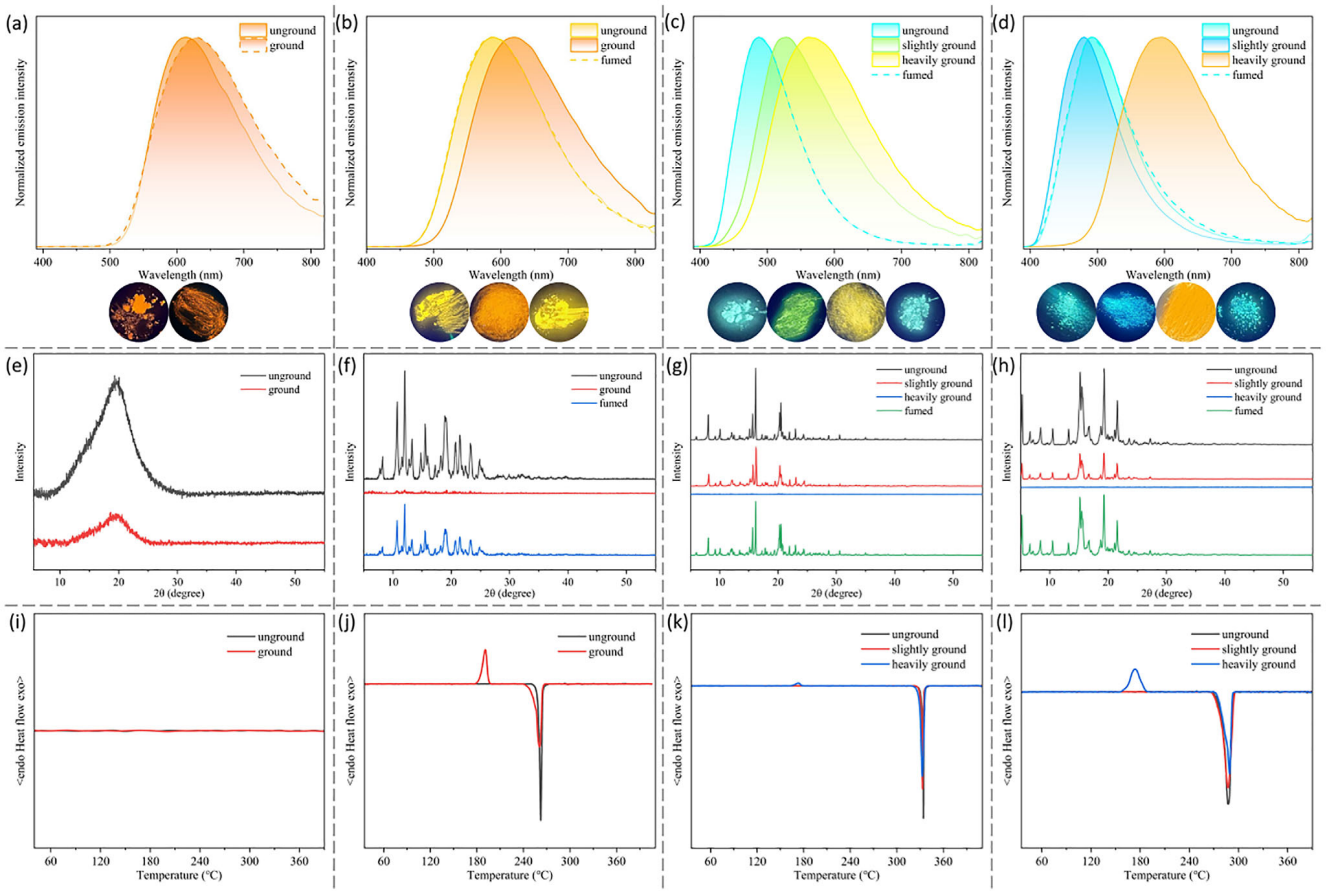
Normalized PL spectra and PL photographs under 365 nm UV excitation of **PhCz3‐*m*‐CF_3_** (a), **PhCz3‐*o*‐CF_3_** (b), **CzPh‐*o*‐CF_3_** (c), and **TPE‐*o*‐CF_3_** (d) in various solid states. Powder XRD patterns of **PhCz3‐*m*‐CF_3_** (e), **PhCz3‐*o*‐CF_3_** (f), **CzPh‐*o*‐CF_3_** (g), and **TPE‐*o*‐CF_3_** (h) in various solid states. DSC curves of **PhCz3‐*m*‐CF_3_** (i), **PhCz3‐*o*‐CF_3_** (j), **CzPh‐*o*‐CF_3_** (k), and **TPE‐*o*‐CF_3_** (l) in various solid states.

The 13 tricolor mechanofluorochromic luminogens were subjected to the final screening stage to determine whether their emission colors underwent a blue‑ or red‑shift upon slight and heavy grinding. The results revealed that most of these luminogens exhibited a gradual red‑shift as the grinding intensity increased. Taking **CzPh‐*o*‐CF_3_** as a representative example (Figure 2c), its pristine solid emitted cyan‑blue fluorescence (*λ*_max_ = 488 nm), which sequentially shifted to yellow‑green (528 nm) upon gentle grinding and to yellow (562 nm) upon vigorous grinding (Figure S8). The original cyan‑blue fluorescence of the sample was recovered after the vigorously ground sample was exposed to CH_2_Cl_2_ vapor. Powder XRD analysis of **CzPh‐*o*‐CF_3_** (Figure 2g) revealed that the as‑prepared powder was highly crystalline, as indicated by its sharp diffraction peaks. Upon mild grinding, these peaks gradually weakened, and they disappeared completely under heavy grinding, indicating a progressive transition to the amorphous phase. Notably, the initial crystalline pattern was readily restored upon solvent fuming, confirming the reversible nature of the morphological transformation. DSC measurements revealed that the pristine sample exhibited a distinct endothermic peak at 334°C, whereas the vigorously ground counterpart showed an additional weak exothermic peak around 170°C. Collectively, these observations indicated that the force‐triggered tricolored fluorescence response of **CzPh‐*o*‐CF_3_** originates from a stepwise phase transformation from a stable crystalline state to an unstable amorphous state. Figures S9–S17 summarize the photophysical data for the other 9 tricolor mechanofluorochromic switches (**Py‐*o*‐CF_3_**, **Py‐*m*‐CF_3_**, **Py‐*p*‐CF_3_**, **CzPh‐*m*‐CF_3_**, **CzPh‐*p*‐CF_3_**, **PhCz2‐H**, **PhCz2‐*o*‐CF**_3_, **PhCz2‐*m*‐CF_3_**, and **PhCz3‐H**), which also exhibited a gradual red‑shift with increasing grinding intensity. Impressively, 3 luminogens, namely **Py‐H**, **TPE‐*o*‐CF_3_**, and **PhCz2‐*p*‐CF_3_**, displayed an extremely rare anisotropic force‐induced hypso‐ and bathochromic bidirectional fluorescence switching. As illustrated in Figure 2d, upon gentle grinding, the initial cyan emission (*λ*_max_ = 493 nm) of solid **TPE‐*o*‐CF_3_** underwent a hypsochromic shift to 480 nm. With further vigorous grinding, the emission shifted to orange‑yellow (*λ*_max_ = 596 nm), representing a substantial red‑shift of 116 nm from the gently ground counterpart (Figure S18). Exposure of the heavily ground solid **TPE‐*o*‐CF_3_** to CH_2_Cl_2_ vapor smoothly restored the original cyan emission, indicative of good reversibility. Powder XRD patterns of **TPE‐*o*‐CF_3_** in various solid states revealed the underlying morphological evolution (Figure 2h). In its pristine state, the compound exhibited sharp diffraction peaks indicative of a well‑ordered crystalline lattice. Upon mild grinding, the intensity of these peaks moderately decreased, suggesting partial loss of crystallinity. After heavy grinding, all sharp diffraction peaks vanished, indicating a complete transition to the amorphous state. CH_2_Cl_2_ vapor treatment readily restored the primal diffraction pattern, consistent with the recovery of the original emission. As exhibited in Figure 2l, for the bidirectional tricolor mechanofluorochromic switch **TPE‐*o*‐CF_3_**, DSC analysis demonstrated stepwise thermal transitions with increasing grinding intensity. The DSC thermograms showed an endothermic peak at approximately 287°C in all three solid states of **TPE‐*o*‐CF_3_**, and the peak intensity diminished with gentle grinding and dropped further upon vigorous grinding. Notably, an additional exothermic peak at 174°C was observed for heavily ground solid **TPE‐*o*‐CF_3_**. Overall, the progressive XRD and DSC variations suggested that the bidirectional tricolor mechanofluorochromic switching of **TPE‐*o*‐CF_3_** was attributable to a stepwise transition, encompassing both crystalline‑to‑amorphous and stable‑to‑unstable transformations, while the phase evolution was effectively governed by grinding intensity. The force‐triggered bidirectional three‐color fluorescence switching characteristics of **Py‐H** and **PhCz2‐*p*‐CF_3_** are also depicted in Figures S19 and S20. The *λ*_max_ values, fluorescence lifetimes, and absolute fluorescence quantum yields of the 20 **R_1_‐R_2_** luminogens in diverse solid states are summarized in Table S1.

### 2.2 Mechanism of Three‐Color Mechanofluorochromism

High‐quality single crystals of **Py‐*o*‐CF_3_** and **TPE‐*o*‐CF_3_** were fortunately obtained (Table S2). As illustrated in Figure 3a and c, the two luminogens are characterized by highly twisted conformations in their crystalline states. The large dihedral angles (Figure S21) between the central dibenzofulvene core and the trifluoromethylbenzene units not only suppressed close π‐π stacking but also promoted loose molecular packing, which was sustained by multiple weak intermolecular interactions. The twisted geometries of **Py‐*o*‐CF_3_** and **TPE‐*o*‐CF_3_** underlay the observed mechanofluorochromic responses. Intermolecular interaction analysis revealed the predominance of C‐H···π and C‐H···F contacts in both crystals (Figure 3b,d). However, compared with **TPE‐*o*‐CF_3_**, **Py‐*o*‐CF_3_** exhibited stronger and more abundant intermolecular interactions (Table S3), which might rigidify its molecular network and render it more resistant to conformational changes under mechanical stress. Meanwhile, projections along the crystallographic axes (Figure 3e,f) revealed substantial intermolecular voids in both crystals, affording sufficient room for conformational changes under external force. **Py‐*o*‐CF_3_** forms an interlocked packing with interdigitated pyrene units, creating a robust network (Figure 3e), whereas **TPE‐*o*‐CF_3_** adopts a herringbone arrangement with staggered packing and no significant interlocking (Figure 3f). This packing divergence directly underlay the distinct force‐triggered fluorescence responses of the two luminogens. For **Py‐*o*‐CF_3_**, the interlocked packing, along with stronger intermolecular interactions, allowed only partial conformational planarization and gradual densification upon grinding, ultimately resulting in tricolor mechanofluorochromism with a progressive red‑shift.

**FIGURE 3:**
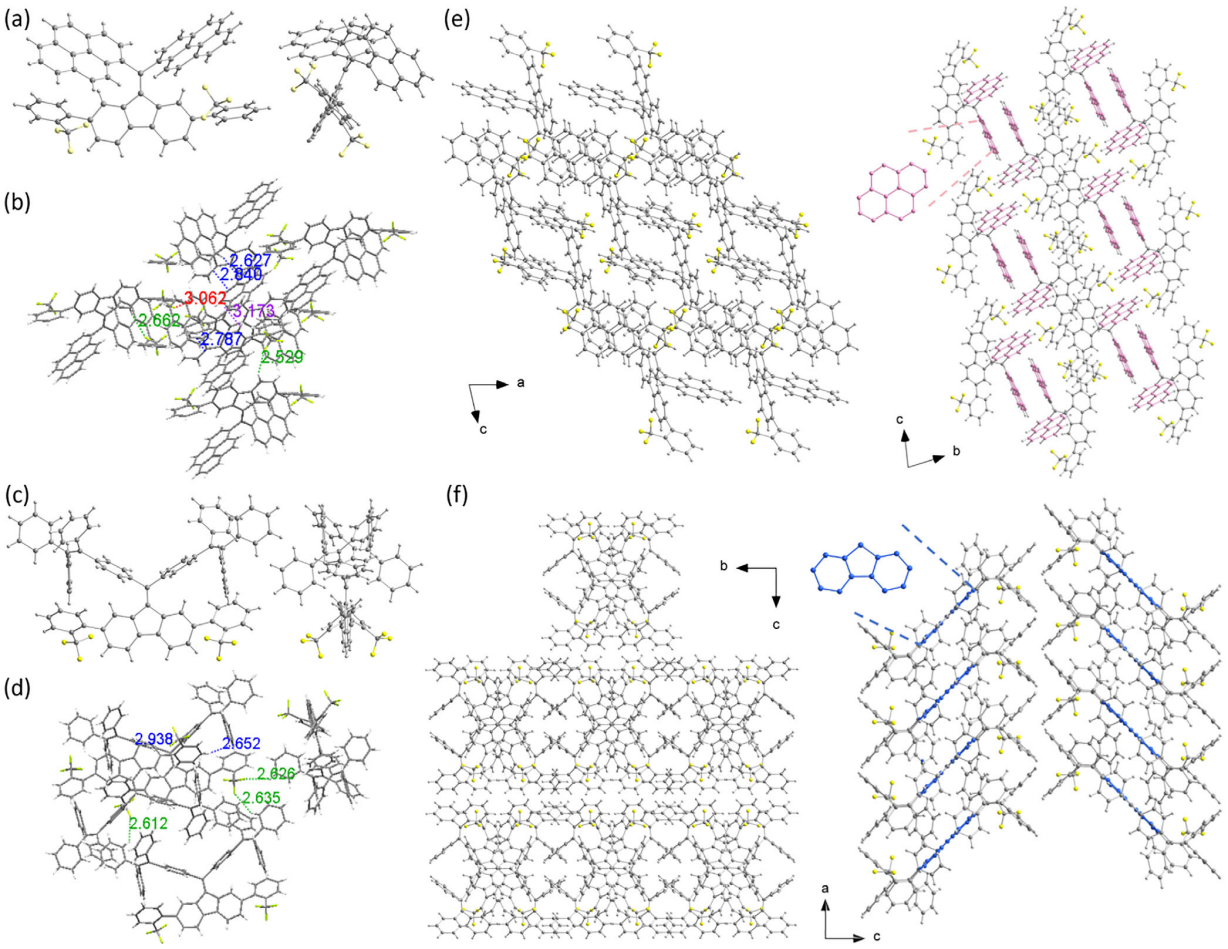
(a) Top and side views of the crystal structure of **Py‐*o*‐CF_3_**. (b) The intermolecular interactions within **Py‐*o*‐CF_3_**: The blue dashed lines are showing intermolecular C‐H∙∙∙π interactions, the green dashed lines are showing intermolecular C‐H∙∙∙F interactions, the purple dashed line is showing intermolecular F∙∙∙π interaction, the red dashed line is showing intermolecular π∙∙∙π interaction. (c) Top and side views of the crystal structure of **TPE‐*o*‐CF_3_**. (d) The intermolecular interactions within **TPE‐*o*‐CF_3_**: The blue dashed lines are showing intermolecular C‐H∙∙∙π interactions, the green dashed lines are showing intermolecular C‐H∙∙∙F interactions. (e) Packing mode of **Py‐*o*‐CF_3_** crystal under different views. (f) Packing mode of **TPE‐*o*‐CF_3_** crystal under different views.

Hirshfeld surface analysis and the corresponding 2D fingerprint plots were generated using CrystalExplorer software to visualize and quantify the intermolecular interactions in the crystals of **Py‐*o*‐CF_3_** and **TPE‐*o*‐CF_3_**. As shown in the Hirshfeld surfaces mapped with the normalized contact distance (*d*_norm_), both crystals displayed red regions, indicative of short contacts shorter than the sum of van der Waals separations, distributed over the molecular surface, primarily attributable to intermolecular C‐H···π and C‐H···F interactions (Figure 4a,b). The 2D fingerprint plots further revealed the detailed characteristics of these interactions (Figure 4c,d and Figure S22). Quantitative analysis of the intermolecular interactions contributions provided clear numerical evidence for the differences between the two crystals (Figure 4e). **Py‐*o*‐CF_3_** possessed a significantly higher proportion of strong intermolecular F···H interactions and a lower proportion of weak intermolecular H···H interactions compared with **TPE‐*o*‐CF_3_**, thus resulting in a more rigid and interconnected crystal lattice. Moreover, crystal void analysis was conducted to quantitatively evaluate the packing efficiency and internal free space within the crystal lattices of **Py‐*o*‐CF_3_** and **TPE‐*o*‐CF_3_** using CrystalExplorer, providing clear visualization of the unoccupied regions. In the crystal of **Py‐*o*‐CF_3_**, isolated cavities are primarily distributed between the interlocked molecular layers (Figure 4f). In contrast, the crystal of **TPE‐*o*‐CF_3_** showcased more extensive, continuous, and interconnected void channels running through the herringbone‐like packing arrangement (Figure 4g). The fractional free volume (FFV) was determined to be 18.4% for **Py‐*o*‐CF_3_** and 21.1% for **TPE‐*o*‐CF_3_**, respectively. Overall, the combined structural analyses indicate that the interlocked packing and stronger directional interactions in **Py‐*o*‐CF_3_** suppress conformational mobility, whereas the herringbone packing and larger FFV in **TPE‐*o*‐CF_3_** facilitate greater molecular reorientation. These contrasting features explain why the two luminogens exhibit unidirectional versus bidirectional fluorescence switching when subjected to ever‑increasing anisotropic forces, respectively. To gain deeper insight into the electronic structure and the origin of the force‐triggered bidirectional tricolor fluorescence switching of **TPE‐*o*‐CF_3_**, density functional theory (DFT) and time‐dependent DFT (TD‐DFT) calculations were carried out for its dimeric form (Figure S23). As depicted in Figure 4h, the frontier molecular orbitals of **TPE‐*o*‐CF_3_** exhibited a distinct spatial separation between the occupied and unoccupied states. The highest occupied molecular orbital (HOMO) and HOMO‑1 were predominantly localized on the tetraphenylethylene donor moieties, while the lowest unoccupied molecular orbital (LUMO) and LUMO+1 were mainly distributed over the trifluoromethylbenzene acceptor units, confirming the intramolecular charge‑transfer (ICT) nature of the molecule. In the pristine state, the cyan emission primarily arose from the S_1_ → S_0_ transition according to the classical Kasha's rule. Upon slight grinding, powder XRD results showed a substantial decrease in crystallinity, whereas the overall molecular conformation remained largely unchanged. The partial disruption of the ordered lattice reduced the rigidity of the crystalline environment and weakened intermolecular interactions. Indeed, gentle grinding likely narrowed the energy gaps between the S_1_, S_2_, and S_3_ excited states. Furthermore, the S_0_ → S_3_ transition exhibited the largest oscillator strength (*f* = 0.03340) among the low‑lying excited states, with the HOMO‐1 → LUMO transition contributing predominantly (91.9%) (Table S4). Therefore, anti‐Kasha emission might occur after slight grinding. In fact, gentle grinding induced a hypsochromic shift of the fluorescence emission, and the observed blue fluorescence was attributed to the S_3_ → S_0_ transition. Upon vigorous grinding, the crystalline structure was completely disrupted, resulting in an amorphous phase. The **TPE‐*o*‐CF_3_** molecule likely underwent significant conformational planarization, which extended the π‑conjugation within the molecular packing and strengthened the ICT character. Consequently, the HOMO‐LUMO gap narrowed significantly, inducing a pronounced bathochromic shift in the fluorescence emission.

**FIGURE 4:**
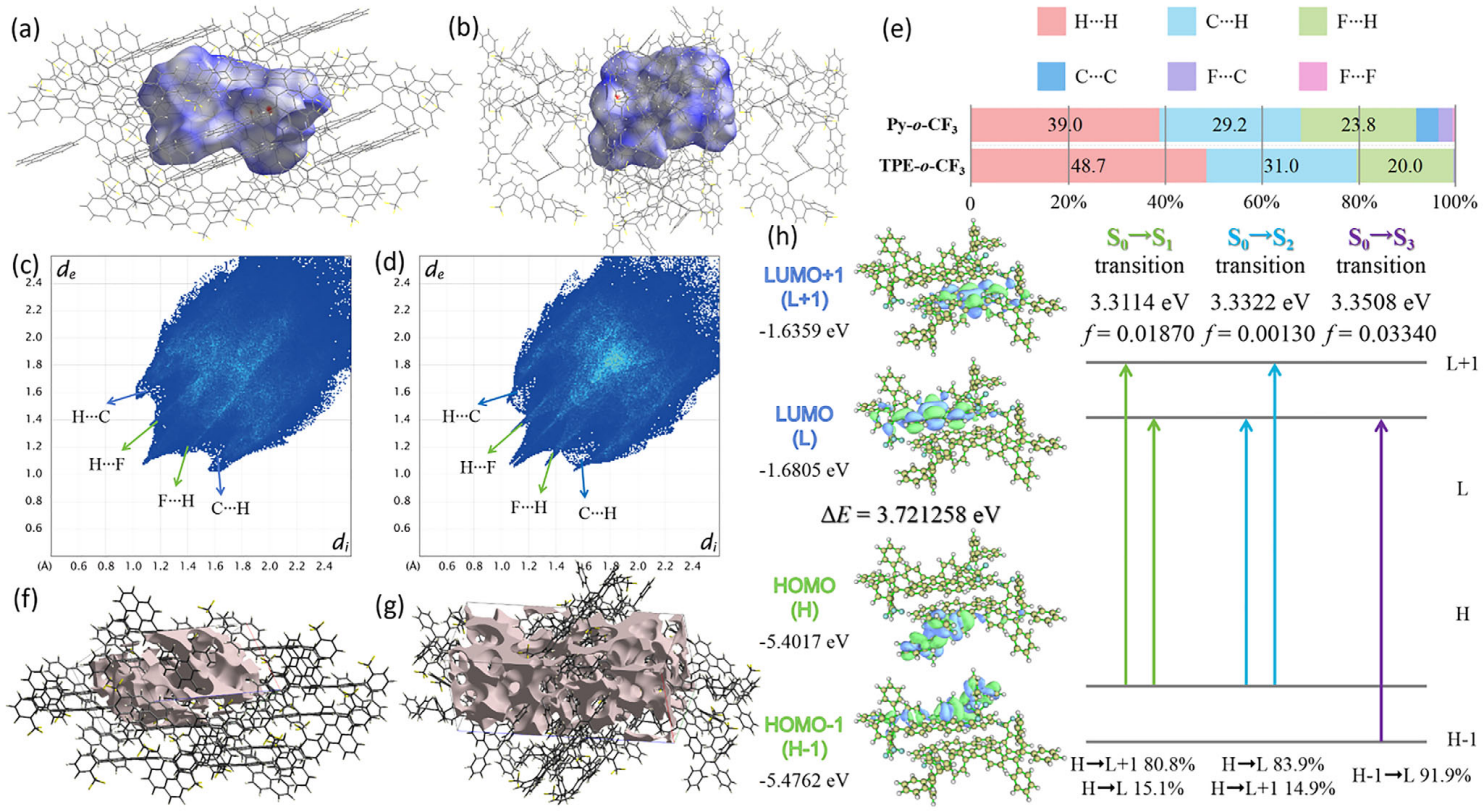
(a) Hirshfeld surface analysis for **Py‐*o*‐CF_3_** mapped with *d*_norm_. (b) Hirshfeld surface analysis for **TPE‐*o*‐CF_3_** mapped with *d*_norm_. (c) 2D fingerprint plot generated by Hirshfeld surface of **Py‐*o*‐CF_3_** crystal. (d) 2D fingerprint plot generated by Hirshfeld surface of **TPE‐*o*‐CF_3_** crystal. (e) Percentage contributions to the Hirshfeld surface area for the various close intermolecular interactions in lumonigens **Py‐*o*‐CF_3_** and **TPE‐*o*‐CF_3_**. (f) Distribution of free volume in the crystal cells of **Py‐*o*‐CF_3_**. (g) Distribution of free volume in the crystal cells of **TPE‐*o*‐CF_3_**. (h) Energy diagrams and the frontier orbital contributions of the **TPE‐*o*‐CF_3_** crystal in its dimeric form.

### 2.3 High‑Security Information Storage Applications

The exceedingly rare bidirectional high‐contrast three‐color mechanofluorochromism exhibited by luminogen **TPE‐*o*‐CF_3_** renders it well‑suited for information storage with a high security level. A dual‑mode information storage platform incorporating ternary coding and quick response (QR) codes was first developed (Figure 5). Pristine **TPE‐*o*‐CF_3_** powder was first patterned into a cyan fluorescent QR code, which could be directly scanned to output the message “tricolor”. Next, gentle grinding turned some cyan‐emitting regions blue, whereas vigorous grinding switched others to orange; the remaining areas retained their original cyan emission. Notably, the two types of fluorescent QR codes obtained via slight grinding followed by vigorous grinding both yielded the same information upon scanning as the pristine one. To enable secondary data storage, a standardized ternary coding protocol was established, in which cyan, blue, and orange fluorescent blocks corresponded to ternary digits “0”, “1”, and “2”, respectively. The full ternary sequence was extracted by reading, in a left‐to‐right, top‐to‐bottom order, the fluorescence signals of the blocks across the patterned QR code, while excluding the three corner position detection patterns and the last four reserved data squares. The hidden message “bidirectional mechanofluorochromic switches” was extracted through subsequent decryption using the predefined 5‐bit ternary mapping table. Integrating visually readable QR codes with high‐capacity ternary data storage, this dual‐layer system provides a reliable and secure information protection paradigm, leveraging a unique anisotropic force‐triggered bidirectional high‐contrast three‐color fluorescence switch.

**FIGURE 5:**
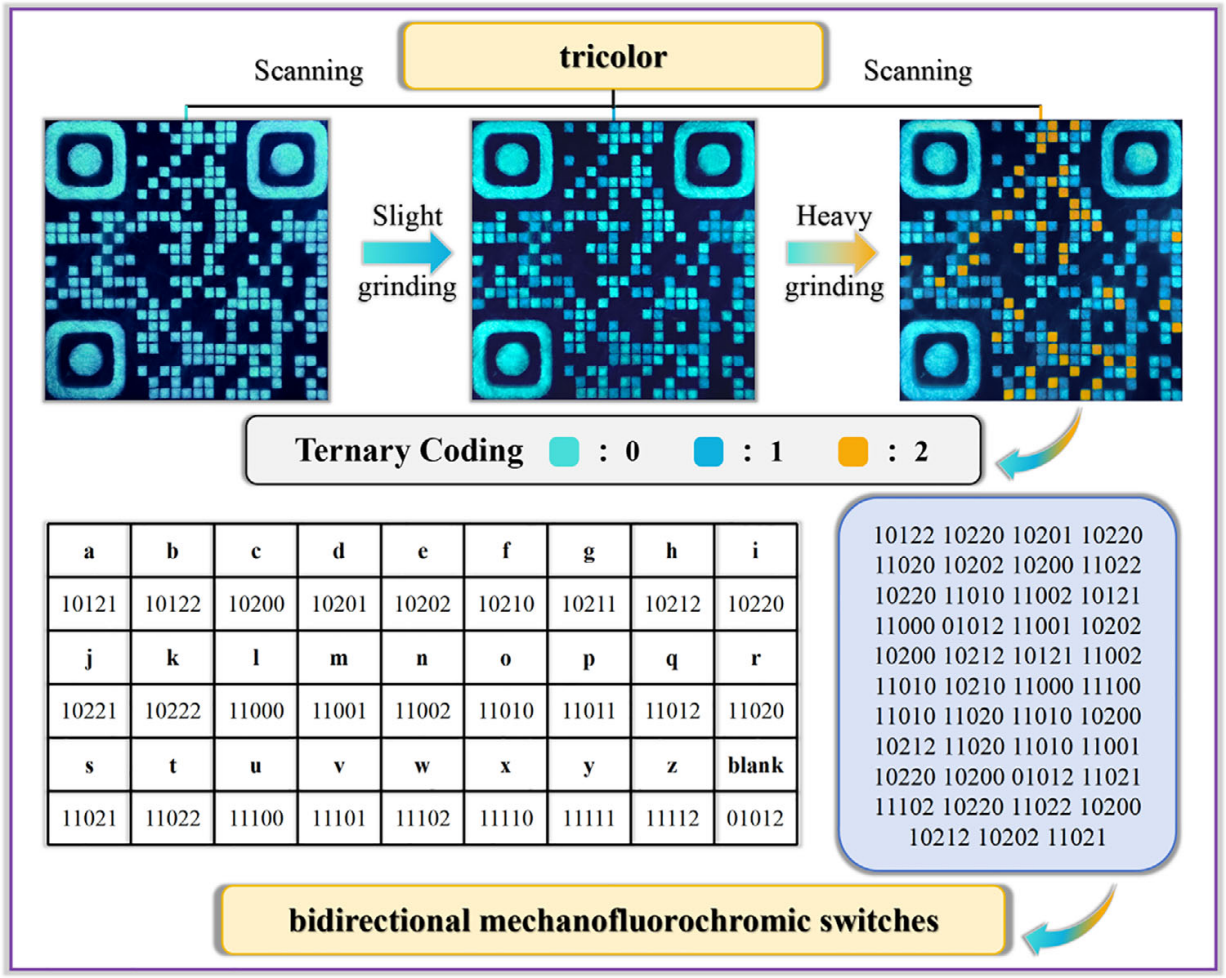
Schematic diagram of the dual‐layer information storage system combining QR codes and ternary coding based on bidirectional tricolor mechanofluorochromism of **TPE‐*o*‐CF_3_**. Three types of QR patterns can be scanned to read the superficial message “tricolor”. Cyan, blue and orange fluorescent blocks are assigned to ternary digits “0”, “1” and “2”, respectively. Decoding the hidden message “bidirectional mechanofluorochromic switches” according to the 5‐bit ternary mapping table.

A second dual‑layer information storage system with a 5×5 layout was constructed using the same bidirectional tricolor mechanofluorochromic luminogen TPE‐*o*‐CF_3_ (Figure 6). Each square of the matrix was filled with the pristine solid TPE‐*o*‐CF_3_ and initially exhibited cyan fluorescence. Regions were then ground either gently or vigorously to selectively convert the emission to blue or orange, respectively, while the unground areas retained the initial cyan luminescence. A ternary coding rule was adopted, whereby the cyan, blue, and orange emission states were mapped to the ternary digits “0”, “1”, and “2”, respectively. Using the 5‑bit ternary character mapping, the luminescent states of all units were read in a left‑to‑right, top‑to‑bottom order, generating a complete ternary string that was subsequently decoded into the first message “JSTNU”. For the secondary storage layer, a dedicated recognition program (available at https://zyj‐color‐code.netlify.app/) was designed to interpret the 5×5 color pattern; the second message “force‐triggered bidirectional tricolor fluorescence switches” was not embedded in the matrix itself but was instead stored within the program and could be successfully extracted only when the pattern was uploaded by the dedicated recognition program and the password “JSTNU” was entered (Figures S24–S26). Benefiting as well from the impressive bidirectional high‐contrast tricolor mechanofluorochromism of TPE‐*o*‐CF_3_, the dual‑layer information storage architecture constructed in this system also offers a high level of security for information protection.

**FIGURE 6:**
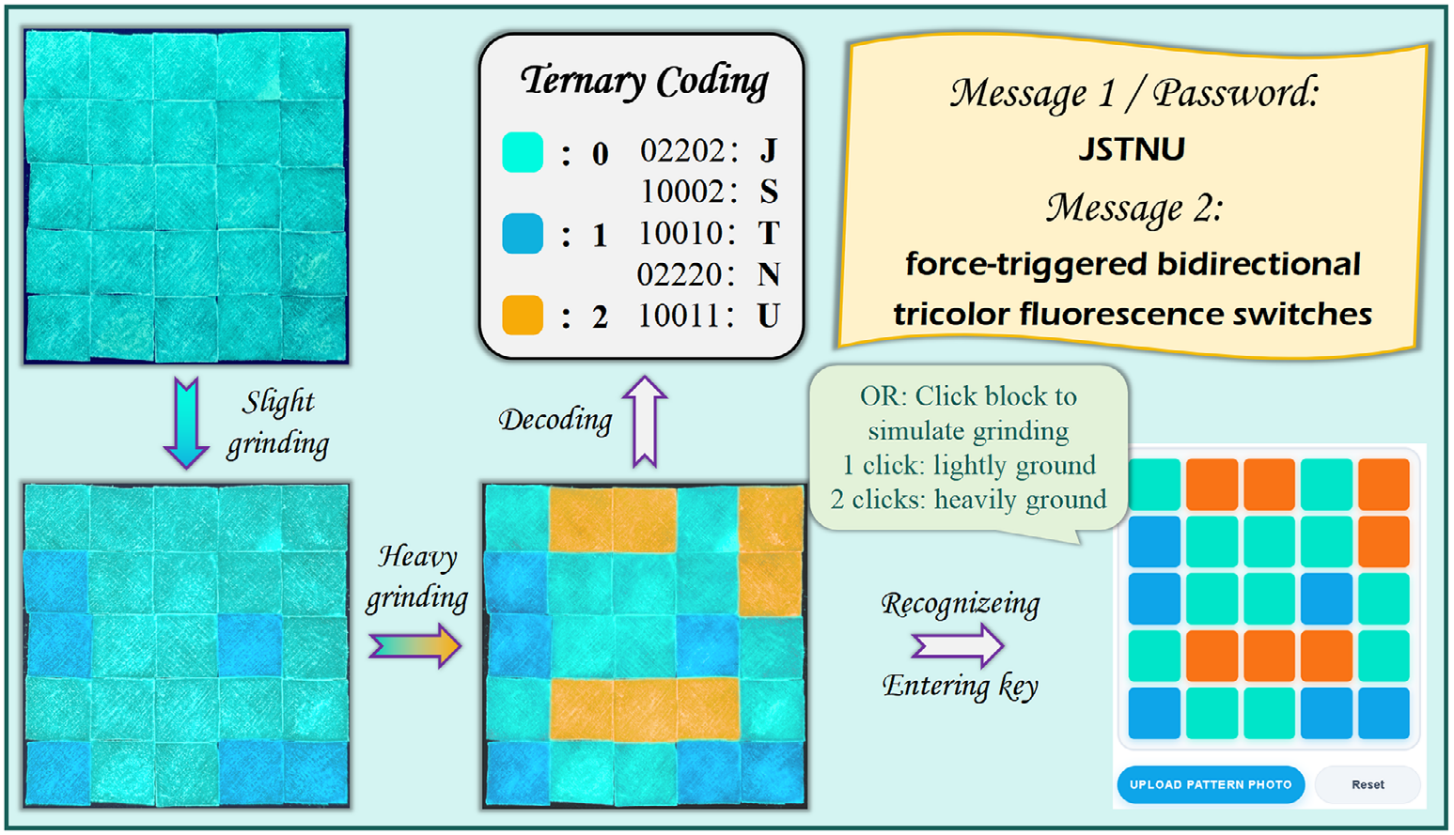
Schematic illustration of color‐coded dual‐layer information storage system based on bidirectional tricolor mechanofluorochromism of **TPE‐*o*‐CF_3_**. Cyan, blue and orange fluorescent blocks are assigned to ternary digits “0”, “1” and “2”, respectively. The 5‑bit ternary string extracted from the matrix in left‑to‑right, top‑to‑bottom order decodes to the message “JSTNU”. Such color code can be identified by a dedicated recognition program, and the hidden message “force‐triggered bidirectional tricolor fluorescence switches” can be obtained after entering the password “JSTNU”.

## 3 Conclusions

In summary, a family of 20 dibenzofulvene‑based luminogens was constructed through a molecular engineering strategy that synergistically integrates substituent variation and positional isomerism, and a highly efficient three‑step screening protocol enabled us to readily obtain 3 exceptionally scarce anisotropic force‐triggered bidirectional three‐color fluorescence‑switching molecules. Specifically, 18 force‐responsive intelligent luminogens were first identified from the 20 prepared dibenzofulvene‑based compounds through the assessment of the mechanoresponsiveness of their solid‑state fluorescence. Subsequently, the feasibility of achieving three‑color fluorescence switching in the obtained 18 luminogens under slight and heavy mechanical grinding was evaluated, and 13 rare three‑color mechanofluorochromic molecular switches were found. Finally, the direction of the emission color changes for the 13 screened force‑responsive three‑color fluorescent switches was examined, yielding 3 exceptionally rare bidirectional mechanofluorochromic luminogens. Mechanistic investigations demonstrated that crystalline‐to‐amorphous progressive phase transitions account for the force‑induced tricolor fluorochromic behaviors. Furthermore, single‑crystal and Hirshfeld surface analyses revealed that the distinct packing modes (interlocked vs. herringbone) and the varying intermolecular interaction networks dictate the divergent mechanofluorochromic properties. Taking advantage of the high‑contrast bidirectional tricolor switching behavior of the best‑performing fluorescent switch TPE‐*o*‐CF_3_, two sophisticated dual‑layer information storage systems combining QR codes, color codes, and ternary encoding were meticulously established, thereby laying a solid foundation for high‑security information protection and validating the practical value of our molecular design strategy. This work not only enriches the family of force‑induced tricolor fluorochromic materials but also provides an effective molecular engineering strategy for the rational construction of novel bidirectional tricolor mechanofluorochromic luminogens, showing promise for advancing the application of force‑triggered three‐color fluorescence switches in high‑security data encryption.

## 4 Experimental Section

### 4.1 Materials/ General Methods/ Instrumentation

The reactive substances 2,7‐diiodo‐9*H*‐fluoren‐9‐one, phenylboronic acid, (2‐(trifluoromethyl)phenyl)boronic acid, (3‐(trifluoromethyl)phenyl)boronic acid, (4‐(trifluoromethyl)phenyl)boronic acid, pyren‐1‐ylboronic acid, (4‐(1,2,2‐triphenylvinyl)phenyl)boronic acid, (4‐(9*H*‐carbazol‐9‐yl)phenyl)boronic acid, (9‐phenyl‐9*H*‐carbazol‐2‐yl)boronic acid, (9‐phenyl‐9*H*‐carbazol‐3‐yl)boronic acid were purchased directly from Innochem (Beijing, China). All the other reactive substances and all reagents were commercially available and used without further purification. All synthetic experiments were carried out under an argon atmosphere applying standard Schlenk techniques. TLC analysis was performed using pre‐coated glass plates. Column chromatography was performed using silica gel (200‐300 mesh). ^1^H NMR and ^13^C NMR spectra were recorded on a Varian INOVA‐500 NMR Spectrometer. ^1^H NMR spectra were reported as followed: chemical shift in ppm (δ) relative to the chemical shift of TMS at 0.00 ppm, integration, multiplicities (s = singlet, d = doublet, t = triplet, m = multiplet), and coupling constant (Hz). ^13^C NMR chemical shifts reported in ppm (δ) relative to the central line of triplet for CDCl_3_ at 77 ppm. Mass spectra were recorded with a Waters G2‐XS Qtof Mass Spectrometer. The X‐ray crystal‐structure determinations of **Py‐*o*‐CF_3_** and **TPE‐*o*‐CF_3_** crystals were carried out on a Bruker APEX DUO CCD system. Photoluminescence spectra were measured by a photoluminescence spectrometer FLS1000 (Edinburgh Instruments). Powder XRD data were recorded on MalvernPanalytical Empyrean diffractometer. DSC data were recorded on Simultaneous Thermal Analyzer (HENVEN, HSC4PLUS). Photographs were recorded on a smart phone (iPhone 14 Pro and Xiaomi 14). The absolute fluorescence quantum yields were measured by a FLS1000 fluorescence spectrophotometer (Edinburgh Instruments) using an integrating sphere and a continuous xenon lamp (450 W) as an excitation source. The fluorescence lifetimes were measured by the FLS1000 spectrofluorometer using a 375 nm EPL picosecond pulsed diode laser.

### 4.2 Theoretical Calculations

Hirshfeld surface, fingerprint plots, crystal void spaces, and energy frameworks were calculated using CrystalExplorer [56, 57, 58]. The DFT and TD‐DFT calculations were performed using the Gaussian 16 program [59]. The optimizations of positions of H atoms in single‐crystal XRD data were performed using B3LYP‐D3BJ [60, 61, 62, 63]/6‐31G* [64, 65]. The TD‐DFT were carried out in the gas phase at the TD‐B3LYP‐D3BJ/6‐31G* level of theory, based on the optimized structures.

### 4.3 Fabrication of Information Storage Applications

The QR code was designed according to IEC 18004, and the corresponding information were read by scanning the QR codes under 365 nm UV light via a smart phone. A QR code pattern encoding the password information was engraved onto a commercially purchased transparent plastic film. The carved scratches create grooves, which were then filled with the mechanofluorochromic powder for subsequent encryption by grinding. Similarly, a 5 × 5 matrix pattern was fabricated by preparing 25 separate small pieces from the same transparent film. Each piece was individually engraved with scratch lines to form grooves. These grooves were then filled with the sample powder. The 25 filled pieces were subsequently arranged into a 5 × 5 array, ready for subsequent grinding encryption. The source codes for image recognition and information decoding used to retrieve the stored data were provided in the Supporting Information. The QR codes and color code recognition program were applied only for exploring anti‐counterfeiting applications and not for any other purposes.

## Author Contributions

**Yijie Zou**: investigation. **Zhao Chen**: writing – original draft, supervision, writing – review and editing, resources. **Shuang Li**: investigation. **Dian‐dian Deng**: investigation. **Hui‐juan Zhang**: investigation. **Hai‐feng He**: supervision. **Ya Yin**: supervision. **Zujin Zhao**: writing – review and editing, supervision.

## Conflicts of Interest

The authors declare no conflicts of interest.

## Supporting information




**Supporting File**: advs78253‐sup‐0001‐SuppMat.docx.

## Data Availability

The data that support the findings of this study are available from the corresponding author upon reasonable request.
