# Supplementary material for: Architecting Anisotropic Force‐Responsive Bidirectional High‐Contrast Three‐Color Fluorescence Switches via Effective Molecular Modulation for High‐Security Information Storage
Source: Adv Sci (Weinh). 2026 Oct 11:e78253. Online ahead of print. doi: 10.1002/advs.78253 (PMC13655248; doi:10.1002/advs.78253)
Supplement: Supplementary file 1 [file ADVS-9999-e78253-s001.docx]

Supporting Information
©Wiley-VCH 2022
69451 Weinheim, Germany

Architecting Anisotropic Force-Responsive Bidirectional High-Contrast Three-Color Fluorescence Switches via Effective Molecular Modulation for High-Security Information Storage

Yijie Zou^1†^, Zhao Chen^1†*^, Shuang Li^1^^†^, Dian-dian Deng^1^, Hui-juan Zhang^1^, Hai-feng He^4*^, Ya Yin^3*^, Zujin Zhao^2*^

^1^	Jiangxi Provincial Key Laboratory of Organic Functional Molecules; Institute of Organic Chemistry, Jiangxi Science and Technology Normal University, Nanchang 330013, P. R. China

^2^	State Key Laboratory of Luminescent Materials and Devices, Guangdong Provincial Key Laboratory of Luminescence from Molecular Aggregates, South China University of Technology, Guangzhou 510640, P. R. China

^3^	State Key Laboratory of Coordination Chemistry, School of Chemistry and Chemical Engineering, Nanjing University, Nanjing 210023, P. R. China

^4^	School of Chemistry and Chemical Engineering, Jiangxi Science and Technology Normal University, Nanchang 330013, P. R. China

Correspondence: Zhao Chen (1020160936@jxstnu.edu.cn); Hai-feng He (hehf0427@jxstnu.com.cn); Ya Yin (yayin@smail.nju.edu.cn); Zujin Zhao (mszjzhao@scut.edu.cn)

^†^ Yijie Zou, Zhao Chen and Shuang Li contributed equally to this work.

Table of Contents

1. Experimental Procedures.
2. Source code for image recognition and information decoding
3. Schemes S1 Synthetic routes of 20 **R_1_-R_2_** compounds.
4. Figures S1-S20 Fluorescence responses and CIE chromaticity diagrams of the luminogens in various solid states before and after grinding.
5. Figure S21 The dihedral angles of **Py-*o*-CF_3_** crystal and **TPE-*o*-CF_3_** crystal
6. Figure S22 2D fingerprint plots of **Py-*o*-CF_3_** crystal and **TPE-*o*-CF_3_** crystal
7. Figure S23. Optimized geometry of the **TPE-*o*-CF_3_** crystal in its dimeric form
8. Figure S24-S26 Color code pattern recognition and data decryption in different states
9. Table S1. The fluorescence parameter of 20 new **R_1_-R_2_** luminogens in different solid states
10. Table S2. Crystal data of **Py-*o*-CF_3_** crystal and **TPE-*o*-CF_3_** crystal
11. Table S3. Multiple intermolecular interactions summarizations of **Py-*o*-CF_3_** crystal and **TPE-*o*-CF_3_** crystal
12. Table S4. Major eclectronic excitations for **TPE-*o*-CF_3_** crystal in its dimeric form
13. Copies of NMR spectra and HRMS spectra of compounds **R_1_-R_2_**
14. References

Experimental Procedures

The synthetic routes of compounds **R_1_-R_2_** are shown in Scheme S1. Compounds **1a-1d** [1,2] and **2a-2d** [3,4] were synthesized by procedures described in the corresponding literatures. Compounds **R_1_-R_2_** were synthesized in a similar manner. Taking the synthetic procedure of **Py-H** as a representative example: A mixture of intermediate **2a** (1.30 mmol), pyren-1-ylboronic acid (2.70 mmol), Na_2_CO_3_ (12.8 mmol), Pd(PPh_3_)_4_ (0.26 mmol) were stirred in THF (80 mL) and H_2_O (8 mL) for two days under an argon atmosphere at 80 ℃. After completion of the reaction, the mixture was extracted with dichloromethane and water, and then washed with brine three times. The combined organic layers were dried over anhydrous MgSO_4_, and concentrated under vacuum. The residual mixture was purified by column chromatography on silica gel using the petroleum ether/dichloromethane as eluent, affording the desired solid product **Py-H** in a good yield of 69%.

**Py-H** (Yellow solid; Yield = 69%): ^1^H NMR (500 MHz, CDCl_3_): δ (ppm) = 8.76 (d, *J* = 10.0 Hz, 1H), 8.65 (d, *J* = 10.0 Hz, 1H), 8.36 (d, *J* = 10.0 Hz, 1H), 8.27-8.24 (m, 3H), 8.21-8.00 (m, 12H), 7.94 (d, *J* = 10.0 Hz, 1H), 7.88 (d, *J* = 10.0 Hz, 1H), 7.42-7.35 (m, 2H), 6.93-6.87 (m, 2H), 6.70 (t, *J* = 7.5 Hz, 2H), 6.60 (t, *J* = 10.0 Hz, 2H), 6.46 (d, *J* = 5.0 Hz, 2H), 6.25 (s, 1H), 6.21 (d, *J* = 5.0 Hz, 1H), 6.11 (s, 1H). ^13^C NMR (125 MHz, CDCl_3_): δ (ppm) = 140.6, 140.4, 139.6, 139.3, 139.2, 139.1, 138.8, 138.4, 138.0, 137.6, 136.3, 131.7, 131.5, 131.4, 131.2, 131.1, 131.0, 130.9, 130.0, 129.6, 129.0, 129.0, 128.9, 128.8, 128.7, 128.1, 128.0, 128.0, 127.6, 127.6, 127.5, 127.5, 127.4, 126.7, 126.7, 126.5, 126.5, 126.4, 126.3, 126.2, 126.1, 126.1, 125.8, 125.8, 125.5, 125.5, 125.4, 125.3, 125.3, 125.3, 125.2, 125.1, 125.0, 124.8, 124.6, 124.5, 124.3, 119.5. HRMS-ESI (m/z): Found: [M + H]^+^ 731.2742; “molecular formula C_58_H_34_” requires [M + H]^+^ 731.2734.

**Py-*o*-CF_3_** (Yellow solid; Yield = 64%): ^1^H NMR (500 MHz, CDCl_3_): δ (ppm) = 8.75 (d, *J* = 10.0 Hz, 2H), 8.28-8.24 (m, 4H), 8.14-8.08 (m, 6H), 8.04 (d, *J* = 10.0 Hz, 2H), 7.98-7.94 (m, 4H), 7.71 (d, *J* = 10.0 Hz, 2H), 7.33(d, *J* = 5.0 Hz, 2H), 7.19 (d, *J* = 10.0 Hz, 2H), 6.96 (t, *J* = 7.5 Hz, 2H), 6.15 (t, *J* = 7.5 Hz, 2H), 5.87 (s, 2H), 5.56 (d, *J* = 5.0 Hz, 2H). ^13^C NMR (125 MHz, CDCl_3_): δ (ppm) = 140.6, 139.6, 138.9, 138.5, 138.1, 138.0, 137.7, 131.7, 131.4, 131.1, 131.0, 130.4, 128.9, 128.9, 128.8, 128.8, 127.8, 127.4, 127.2, 126.6, 126.5, 126.2, 126.2, 125.7, 125.7, 125.5, 125.3, 125.2, 125.0, 118.5. HRMS-ESI (m/z): Found: [M + H]^+^ 867.2490; “molecular formula C_60_H_32_F_6_” requires [M + H]^+^ 867.2481.

**Py-*m*-CF_3_** (Yellow solid; Yield = 66%): ^1^H NMR (500 MHz, CDCl_3_): δ (ppm) = 8.74 (d, *J* = 10.0 Hz, 2H), 8.30 (d, *J* = 5.0 Hz, 2H), 8.20-8.16 (m, 6H), 8.13-8.05 (m, 8H), 7.79 (d, *J* = 10.0 Hz, 2H), 7.41 (d, *J* = 5.0 Hz, 2H), 7.17(d, *J* = 5.0 Hz, 2H), 6.89 (s, 2H), 6.64 (t, *J* = 7.5 Hz, 2H), 6.20 (d, *J* = 7.5 Hz, 4H). ^13^C NMR (125 MHz, CDCl_3_): δ (ppm) = 141.4, 141.2, 139.9, 139.9, 139.4, 138.8, 138.1, 138.0, 137.8, 137.6, 137.2, 131.8, 131.6, 131.5, 131.3, 131.0, 130.8, 130.7, 130.4, 129.6, 129.5, 129.4, 129.1, 128.8, 128.7, 128.6, 128.5, 128.5, 128.3, 128.2, 127.3, 126.9, 126.4, 126.3, 126.1, 126.1, 125.7, 125.7, 125.7, 125.5, 125.3, 125.2, 125.2, 125.1, 125.1, 124.9, 124.9, 124.7, 124.5, 124.5, 124.1, 123.3, 123.2, 122.7, 122.7, 122.6, 119.9. HRMS-ESI (m/z): Found: [M + H]^+^ 867.2485; “molecular formula C_60_H_32_F_6_” requires [M + H]^+^ 867.2481.

**Py-*p*-CF_3_** (Yellow solid; Yield = 67%): ^1^H NMR (500 MHz, CDCl_3_): δ (ppm) = 8.72 (d, *J* = 10.0 Hz, 2H), 8.35 (d, *J* = 5.0 Hz, 2H), 8.25-8.18 (m, 6H), 8.13-8.08 (m, 8H), 7.76 (d, *J* = 5.0 Hz, 2H), 7.37 (d, *J* = 10.0 Hz, 2H), 6.85(d, *J* = 5.0 Hz, 4H), 6.29 (d, *J* = 10.0 Hz, 4H), 6.07 (s, 2H). ^13^C NMR (125 MHz, CDCl_3_): δ (ppm) = 143.9, 140.0, 139.8, 138.6, 138.1, 137.9, 137.6, 131.5, 131.3, 131.0, 129.1, 129.0, 128.4, 128.2, 127.4, 126.9, 126.6, 126.3, 126.3, 125.8, 125.7, 125.4, 125.1, 125.1, 125.0, 125.0, 124.9, 124.4, 119.8. HRMS-ESI (m/z): Found: [M + Na]^+^ 889.2302; “molecular formula C_60_H_32_F_6_” requires [M + Na]^+^ 889.2301.

**TPE-H** (Orange-yellow solid; Yield = 66%): ^1^H NMR (500 MHz, CDCl_3_): δ (ppm) = 7.77 (d, *J* = 5.0 Hz, 2H), 7.51-7.42 (m, 10H), 7.39-7.36(m, 2H), 7.27 (d, *J* = 1.0 Hz, 2H), 7.13-7.00(m, 34H), 6.87 (t, *J* = 5.0 Hz, 4H). ^13^C NMR (125 MHz, CDCl_3_): δ (ppm) = 146.1, 144.0, 143.7, 143.6 143.4, 141.9, 141.7, 141.2, 140.4, 139.8, 139.4, 139.2, 133.4, 131.7, 131.5, 131.4, 129.9, 128.8, 127.8, 127.7, 127.6, 127.1, 127.0, 126.9, 126.6, 126.5, 123.8, 119.5. HRMS-ESI (m/z): Found: [M + H]^+^ 991.4301; “molecular formula C_78_H_54_” requires [M + H]^+^ 991.4299.

**TPE-*o*-CF_3_** (Light yellow solid; Yield = 62%): ^1^H NMR (500 MHz, CDCl_3_): δ (ppm) = 7.79 (d, *J* = 10.0 Hz, 2H), 7.75 (d, *J* = 5.0 Hz, 2H), 7.60 (t, *J* = 7.5 Hz, 2H), 7.52 (t, *J* = 7.5 Hz, 2H), 7.28 (d, *J* = 10.0 Hz, 2H), 7.23 (d, *J* = 10.0 Hz, 2H), 7.11-7.06 (m, 8H), 7.02-6.91 (m, 20H), 6.88-6.84 (m, 8H), 6.76 (t, *J* = 7.5 Hz, 4H). ^13^C NMR (125 MHz, CDCl_3_): δ (ppm) = 146.2, 143.9, 143.5, 143.5, 143.3, 142.1, 141.4, 140.8, 140.5, 139.2, 138.7, 137.9, 133.2, 132.1, 131.5, 131.3, 131.3, 129.8, 128.6, 128.5, 128.4, 127.7, 127.6, 127.4, 127.3 126.5, 126.5, 126.4, 126.1, 126.1, 125.5, 125.2, 123.0, 118.5. HRMS-ESI (m/z): Found: [M + H]^+^ 1127.4048; “molecular formula C_80_H_52_F_6_” requires [M + H]^+^ 1127.4046.

**TPE-*m*-CF_3_** (Light yellow solid; Yield = 63%): ^1^H NMR (500 MHz, CDCl_3_): δ (ppm) = 7.82 (d, *J* = 5.0 Hz, 2H), 7.73 (s, 2H), 7.64-7.59 (m, 6H), 7.50-7.48 (m, 2H), 7.12-7.02 (m, 30H), 6.98-6.94 (m, 6H), 6.77 (t, *J* = 10.0 Hz, 4H). ^13^C NMR (125 MHz, CDCl_3_): δ (ppm) = 141.4, 141.2, 139.9, 139.9, 139.5, 139.8, 138.1, 138.0, 137.8, 137.7, 137.3, 131.8, 131.6, 131.3, 131.0, 130.7, 130.4, 129.5, 129.1, 128.8, 128.8, 128.6, 128.5, 128.5, 128.3, 128.2, 127.6, 127.5, 127.4, 126.9, 126.4, 126.3, 126.2, 125.7, 125.7, 125.5, 125.3, 125.2, 125.1, 125.1, 125.0, 124.9, 124.7, 124.5, 124.5, 124.1, 123.2, 122.7, 122.7, 119.9. HRMS-ESI (m/z): Found: [M + H]^+^ 1127.4058; “molecular formula C_80_H_52_F_6_” requires [M + H]^+^ 1127.4046.

**TPE-*p*-CF_3_** (Light yellow solid; Yield = 70%): ^1^H NMR (500 MHz, CDCl_3_): δ (ppm) = 7.81 (d, *J* = 10.0 Hz, 2H), 7.71 (d, *J* = 10.0 Hz, 4H), 7.51 (d, *J* = 5.0 Hz, 6H), 7.29 (s, 2H), 7.15-7.11 (m, 8H), 7.08-7.02 (m, 22H), 6.99 (d, *J* = 5.0 Hz, 4H), 6.92 (t, *J* = 7.5 Hz, 4H). ^13^C NMR (125 MHz, CDCl_3_): δ (ppm) = 147.0, 145.3, 144.3, 143.5, 143.4, 143.2, 142.1, 140.9, 140.2, 140.0, 139.6, 138.0, 132.8, 131.6, 131.3, 131.3, 129.9, 127.8, 127.8, 127.7, 127.6, 127.2, 126.9, 126.7, 126.6, 125.7, 125.7, 123.9, 119.9.RMS-ESI (m/z): Found: [M + H]^+^ 1127.4059; “molecular formula C_80_H_52_F_6_” requires [M + H]^+^ 1127.4046.

**CzPh-H** (Orange solid; Yield = 68%): ^1^H NMR (500 MHz, CDCl_3_): δ (ppm) = 9.47 (d, *J* = 5.0 Hz, 4H), 9.15-9.09 (m, 6H), 9.04 (d, *J* = 10.0 Hz, 4H), 8.91 (d, *J* = 10.0 Hz, 2H), 8.78 (d, *J* = 5.0 Hz, 4H), 8.71-8.68 (m, 8H), 8.68 (t, *J* = 7.5 Hz, 4H), 8.57-8.52 (m, 8H). ^13^C NMR (125 MHz, CDCl_3_): δ (ppm) = 146.9, 144.5, 143.9, 143.6, 142.6, 142.5, 142.3, 141.0, 138.5, 134.8, 132.2, 130.4, 130.4, 130.3, 129.6, 129.4, 126.9, 126.4, 123.5, 123.5, 123.2, 113.1. HRMS-ESI (m/z): Found: [M + K]^+^ 851.2820; “molecular formula C_62_H_40_N_2_” requires [M + K]^+^ 851.2824.

**CzPh-*o*-CF_3_** (Oyster white solid; Yield = 61%): ^1^H NMR (500 MHz, CDCl_3_): δ (ppm) = 8.13 (d, *J* = 10.0 Hz, 4H), 7.83 (d, *J* = 10.0 Hz, 2H), 7.74 (d, *J* = 5.0 Hz, 6H), 7.60 (d, *J* = 10.0 Hz, 4H), 7.49-7.42 (m, 5H), 7.33-7.27 (m, 12H), 7.15 (s, 3H), 6.91 (s, 2H). ^13^C NMR (125 MHz, CDCl_3_): δ (ppm) = 147.3, 144.6, 144.3, 143.6, 142.6, 141.4, 141.2, 140.6, 138.1, 134.9, 134.7, 134.4, 132.3, 131.2, 131.0, 130.6, 130.5, 129.3, 129.1, 129.1, 128.9, 128.4, 126.2, 126.2, 123.4, 123.3, 122.1, 112.7. HRMS-ESI (m/z): Found: [M + H]^+^ 949.3021; “molecular formula C_64_H_38_F_6_N_2_” requires [M + H]^+^ 949.3012.

**CzPh-*m*-CF_3_** (Pale yellow solid; Yield = 66%): ^1^H NMR (500 MHz, CDCl_3_): δ (ppm) = 8.18 (t, *J* = 5.0 Hz, 4H), 7.90 (d, *J* = 10.0 Hz, 2H), 7.82 (d, *J* = 10.0 Hz, 4H), 7.77 (t, *J* = 7.5 Hz, 6H), 7.62 (d, *J* = 10.0 Hz, 2H), 7.46-7.43 (m, 8H), 7.35-7.31 (m, 8H), 7.22 (t, *J* = 7.5 Hz, 4H). ^13^C NMR (125 MHz, CDCl_3_): δ (ppm) = 144.0, 141.6, 141.4, 140.7, 139.7, 138.3, 138.2, 137.7, 135.2, 131.7, 131.4, 131.3, 129.1, 128.5, 128.2, 127.6, 127.4, 126.0, 125.8, 125.2, 123.4, 120.2, 120.0, 119.0, 109.6. HRMS-ESI (m/z): Found: [M + H]^+^ 949.3010; “molecular formula C_64_H_38_F_6_N_2_” requires [M + H]^+^ 949.3012.

**CzPh-*p*-CF_3_** (Light yellow solid; Yield = 71%): ^1^H NMR (500 MHz, CDCl_3_): δ (ppm) = 8.21-8.19 (m, 4H), 7.90 (d, *J* = 5.0 Hz, 2H), 7.83-7.74 (m, 9H), 7.64-7.62 (m, 2H), 7.50-7.43 (m, 12H), 7.39-7.33 (m, 9H). ^13^C NMR (125 MHz, CDCl_3_): δ (ppm) = 144.5, 141.9, 141.1, 140.5, 139.9, 139.6, 138.4, 138.2, 135.2, 131.5, 131.3, 131.0, 129.6, 129.5, 127.4, 127.3, 126.1, 125.1, 124.0, 123.8, 123.6, 120.4, 120.4, 120.3, 109.7. HRMS-ESI (m/z): Found: [M + H]^+^ 949.3002; “molecular formula C_64_H_38_F_6_N_2_” requires [M + H]^+^ 949.3012.

**PhCz2-H** (Pale yellow solid; Yield = 70%): ^1^H NMR (500 MHz, CD_2_Cl_2_): δ (ppm) = 8.97 (d, *J* = 5.0 Hz, 2H), 8.08-8.04 (m, 4H), 7.76 (d, *J* = 10.0 Hz, 2H), 7.65 (t, *J* = 7.5 Hz, 2H), 7.52 (t, *J* = 7.5 Hz, 2H), 7.16-7.11 (m, 15H), 7.10-7.06 (m, 11H), 7.03 (s, 2H). ^13^C NMR (125 MHz, CDCl_3_): δ (ppm) = 148.3, 146.9, 143.6, 143.0, 143.0, 142.2, 141.5, 137.4, 135.7, 134.5, 131.4, 130.6, 130.5, 130.4, 129.2, 129.0, 128.0, 127.9, 127.8, 127.8, 127.7, 127.7, 127.7, 127.6, 127.1, 126.8, 126.7, 126.6. HRMS-ESI (m/z): Found: [M + H]^+^ 813.3269; “molecular formula C_62_H_40_N_2_” requires [M + H]^+^ 813.3265.

**PhCz2-*o*-CF_3_** (Yellow solid; Yield = 64%): ^1^H NMR (500 MHz, CDCl_3_): δ (ppm) = 8.09-8.03 (m, 4H), 7.74-7.72 (m, 2H), 7.60 (s, 1H), 7.53-7.34 (m, 18H), 7.28 (d, *J* = 5.0 Hz, 1H), 7.21 (d, *J* = 5.0 Hz, 4H), 7.16-7.05 (m, 4H), 6.92 (d, *J* = 10.0 Hz, 1H), 6.86 (d, *J* = 10.0 Hz, 1H), 6.68 (s, 2H). ^13^C NMR (125 MHz, CDCl_3_): δ (ppm) = 149.4, 145.0, 141.5, 141.3, 141.3, 140.5, 139.4, 137.6, 137.6, 137.4, 134.7, 134.7, 133.3, 130.1, 129.1, 128.9, 128.8, 128.5, 127.9, 127.1, 127.0, 126.5, 126.5, 126.0, 125.3, 125.3, 125.2, 124.1, 124.0, 123.9, 123.2, 123.1, 123.1, 123.0, 120.6, 120.5, 120.5, 119.8, 110.1, 110.0, 109.9.

HRMS-ESI (m/z): Found: [M + H]^+^ 949.3019; “molecular formula C_64_H_38_F_6_N_2_” requires [M + H]^+^ 949.3012.

**PhCz2-*m*-CF_3_** (Yellow solid; Yield = 64%): ^1^H NMR (500 MHz, CDCl_3_): δ (ppm) = 8.26-8.18 (m, 4H), 7.79 (d, *J* = 5.0 Hz, 2H), 7.61 (s, 1H), 7.50-7.30 (m, 25H), 7.13-7.08 (m, 2H), 6.96-6.90 (m, 2H), 6.84 (d, *J* = 10.0 Hz, 2H). ^13^C NMR (125 MHz, CDCl_3_): δ (ppm) = 148.6, 148.3, 141.5, 141.4, 140.9, 140.8, 140.8, 139.2, 139.1, 139.1, 138.0, 137.3, 134.0, 133.9, 131.8, 131.7, 131.0, 129.9, 129.8, 128.5, 128.1, 128.0, 127.9, 127.3, 126.9, 126.9, 126.8, 126.1, 126.1, 126.1, 126.0, 125.5, 125.4, 125.2, 123.4, 123.1, 123.1, 123.0, 122.5, 122.1, 120.6, 120.4, 120.0, 118.5, 112.0, 111.6, 109.8, 109.8. HRMS-ESI (m/z): Found: [M + H]^+^ 949.3019; “molecular formula C_64_H_38_F_6_N_2_” requires [M + H]^+^ 949.3012.

**PhCz2-*p*-CF_3_** (Yellowish white solid; Yield = 66%): ^1^H NMR (500 MHz, CDCl_3_): δ (ppm) = 8.25-8.19 (m, 4H), 7.78-7.76 (m, 2H), 7.67 (s, 1H), 7.52-7.33 (m, 21H), 7.13-7.05 (m, 8H), 6.72 (d, *J* = 15.0 Hz, 2H). ^13^C NMR (125 MHz, CDCl_3_): δ (ppm) = 148.9, 148.7, 141.7, 141.7, 141.6, 141.1, 140.2, 140.2, 140.0, 139.5, 137.5, 137.3, 137.3, 134.1, 134.1, 131.0, 130.7, 129.9, 129.8, 129.5, 128.9, 127.5, 127.4, 126.9, 126.4, 126.2, 123.9, 123.9, 123.8, 123.8, 123.5, 123.4, 123.2, 123.2, 123.1, 123.1, 123.0, 123.0, 122.8, 122.0, 121.8, 120.8, 120.7, 120.6, 120.6, 120.3, 119.9, 111.5, 111.2, 109.9, 109.8, HRMS-ESI (m/z): Found: [M + H]^+^ 949.3016; “molecular formula C_64_H_38_F_6_N_2_” requires [M + H]^+^ 949.3012.

**PhCz3-H** (Light yellow solid; Yield = 58%): ^1^H NMR (500 MHz, CDCl_3_): δ (ppm) = 8.40 (s, 1H), 8.31 (s, 1H), 8.11 (d, *J* = 5.0 Hz, 1H), 8.06 (d, *J* = 5.0 Hz, 1H), 7.81 (d, *J* = 10.0 Hz, 2H), 7.69 (d, *J* = 10.0 Hz, 1H), 7.66-7.58 (m, 9H), 7.52-7.41 (m, 10H), 7.28 (d, *J* = 5.0 Hz, 1H), 7.24 (d, *J* = 5.0 Hz, 1H), 7.15-7.10 (m, 6H), 7.06-7.01 (m, 6H). ^13^C NMR (125 MHz, CDCl_3_): δ (ppm) = 148.5, 141.5, 141.4, 141.2, 141.2, 140.3, 139.0, 138.8, 138.8, 137.6, 135.0, 135.0, 133.7, 130.0, 129.3, 129.1, 128.3, 127.7, 127.1, 126.7, 126.6, 126.3, 126.0, 123.9, 123.8, 123.8, 123.4, 123.2, 123.0, 120.7, 120.4, 120.3, 119.5, 110.0, 109.9. HRMS-ESI (m/z): Found: [M + H]^+^ 813.3268; “molecular formula C_62_H_40_N_2_” requires [M + H]^+^ 813.3265.

**PhCz3-*o*-CF_3_** (Yellow solid; Yield = 52%): ^1^H NMR (500 MHz, CDCl_3_): δ (ppm) = 8.28 (s, 1H), 8.20 (s, 1H), 8.06 (d, *J* = 5.0 Hz, 1H), 8.01 (d, *J* = 5.0 Hz, 1H), 7.80 (d, *J* = 10.0 Hz, 2H), 7.63-7.55 (m, 8H), 7.51-7.31 (m, 15H), 7.24-7.22 (m, 5H), 6.95-6.90 (m, 2H), 6.75 (s, 2H). ^13^C NMR (125 MHz, CDCl_3_): δ (ppm) = 149.0, 141.8, 141.3, 141.2, 141.1, 139.5, 139.0, 137.8, 137.8, 137.4, 135.0, 133.0, 132.3, 132.3, 130.7, 129.9, 129.6, 127.9, 127.6, 127.0, 126.8, 126.1, 125.8, 125.8, 125.7, 125.5, 125.2, 123.6, 123.6, 123.6, 123.5, 123.4, 123.0, 120.6, 120.3, 120.2, 118.4, 109.9, 109.8, 109.8. HRMS-ESI (m/z): Found: [M + H]^+^ 949.3019; “molecular formula C_64_H_38_F_6_N_2_” requires [M + H]^+^ 949.3012.

**PhCz3-*m*-CF_3_** (Orange solid; Yield = 61%): ^1^H NMR (500 MHz, CDCl_3_): δ (ppm) = 8.35 (d, *J* = 5.0 Hz, 2H), 8.09-8.04 (m, 2H), 7.88 (d, *J* = 5.0 Hz, 2H), 7.65-7.55 (m, 11H), 7.52-7.41 (m, 13H), 7.36 (d, *J* = 10.0 Hz, 2H), 7.24 (d, *J* = 5.0 Hz, 2H), 7.13-7.08 (m, 4H). ^13^C NMR (125 MHz, CDCl_3_): δ (ppm) = 149.7, 149.6, 142.2, 141.5, 141.2, 141.2, 140.7, 139.3, 137.6, 137.4, 134.8, 134.8, 132.9, 132.8, 131.2, 130.9, 130.7, 130.4, 130.0, 129.9, 129.0, 128.7, 127.7, 127.1, 126.4, 126.0, 125.0, 123.8, 123.7, 123.3, 123.2, 122.8, 120.5, 120.4, 120.4, 119.9, 110.0, 110.0, 110.0. HRMS-ESI (m/z): Found: [M + H]^+^ 949.3014; “molecular formula C_64_H_38_F_6_N_2_” requires [M + H]^+^ 949.3012.

**PhCz3-*p*-CF_3_** (Orange yellow solid; Yield = 62%): ^1^H NMR (500 MHz, CDCl_3_): δ (ppm) = 8.39, (s, 1H), 8.30 (s, 1H), 8.11 (d, *J* = 10.0 Hz, 1H), 8.06 (d, *J* = 10.0 Hz, 1H), 7.84 (d, *J* = 10.0 Hz, 2H), 7.70-7.63 (m, 5H), 7.59-7.45 (m, 15H), 7.29 (t, *J* = 10.0 Hz, 6H), 7.19 (t, *J* = 7.5 Hz, 4H), 6.93 (s, 2H). ^13^C NMR (125 MHz, CDCl_3_): δ (ppm) = 149.4, 144.9, 141.5, 141.3, 141.3, 140.5, 139.4, 137.6, 137.5, 137.4, 134.7, 134.7, 133.3, 130.0, 129.1, 128.8, 128.7, 128.5, 127.9, 127.0, 126.9, 126.5, 126.0, 125.3, 125.2, 125.2, 124.0, 124.0, 123.9, 123.2, 123.1, 123.1, 123.0, 120.6, 120.5, 120.5, 119.9, 110.1, 110.0, 109.9. HRMS-ESI (m/z): Found: [M + H]^+^ 949.3018; “molecular formula C_64_H_38_F_6_N_2_” requires [M + H]^+^ 949.3012.

**Source code for image recognition and information decoding**

<!DOCTYPE html>

<html lang="en">

<head>

<meta charset="UTF-8">

<meta name="viewport" content="width=device-width, initial-scale=1.0">

<title>COLOR CODE - 5x5 Pattern Recognition and Data Decryption</title>

<!-- Introduce OpenCV.js for precise contour positioning and grid recognition -->

<script async src="https://docs.opencv.org/4.8.0/opencv.js" onload="onCvReady()"></script>

<style>

:root {

--bg-light: #f0f4f9;

--bg-gradient: radial-gradient(circle at 50% 0%, #ffffff 0%, #e2e8f0 100%);

--card-bg: #ffffff;

--panel-bg: #f8fafc;

--input-bg: #ffffff;

--border-color: #e2e8f0;

--cyan-neon: #00e5c9;

--blue-neon: #0ea5e9;

--orange-neon: #f97316;

--text-main: #0f172a;

--text-muted: #64748b;

--primary-color: #0ea5e9;

--primary-hover: #0284c7;

}

* { box-sizing: border-box; margin: 0; padding: 0; }

body {

font-family: -apple-system, BlinkMacSystemFont, "Segoe UI", Roboto, Oxygen, Ubuntu, Cantarell, sans-serif;

background: var(--bg-gradient);

color: var(--text-main);

min-height: 100vh;

display: flex;

justify-content: center;

align-items: center;

padding: 32px 20px;

}

.app-container {

background: var(--card-bg);

border: 1px solid rgba(226, 232, 240, 0.9);

border-radius: 28px;

width: 100%;

max-width: 940px;

padding: 40px 48px;

box-shadow: 0 20px 40px -15px rgba(15, 23, 42, 0.07), 0 0 1px 1px rgba(0, 0, 0, 0.02);

}

/* Header & Brand */

.header-logo {

display: flex;

align-items: center;

gap: 12px;

margin-bottom: 24px;

}

.logo-icon {

width: 28px;

height: 28px;

display: grid;

grid-template-columns: repeat(3, 1fr);

gap: 3px;

}

.dot-c { background: var(--cyan-neon); border-radius: 3px; }

.dot-b { background: var(--blue-neon); border-radius: 3px; }

.dot-o { background: var(--orange-neon); border-radius: 3px; }

.logo-text h4 { font-size: 15px; font-weight: 800; letter-spacing: 1.8px; margin: 0; color: #0f172a; }

.logo-text p { font-size: 10px; color: var(--blue-neon); letter-spacing: 2px; margin: 0; font-weight: 700; }

.main-title {

font-size: 26px;

font-weight: 700;

text-align: center;

margin-bottom: 36px;

letter-spacing: -0.5px;

color: #0f172a;

}

.two-column-layout {

display: grid;

grid-template-columns: 1.05fr 0.95fr;

gap: 38px;

}

@media (max-width: 768px) {

.two-column-layout { grid-template-columns: 1fr; gap: 28px; }

.app-container { padding: 28px 20px; }

}

.step-title {

font-size: 12px;

font-weight: 700;

color: var(--text-muted);

letter-spacing: 1.2px;

text-transform: uppercase;

margin-bottom: 14px;

}

/* 5x5 Main Grid Container */

.grid-container {

width: 100%;

max-width: 320px;

aspect-ratio: 1 / 1;

margin: 0 auto 16px auto;

border: 2px solid #e2e8f0;

border-radius: 18px;

background: #f8fafc;

padding: 10px;

display: grid;

grid-template-columns: repeat(5, 1fr);

gap: 6px;

box-shadow: inset 0 2px 4px rgba(0, 0, 0, 0.03);

}

.cell {

border-radius: 6px;

cursor: pointer;

transition: all 0.18s cubic-bezier(0.4, 0, 0.2, 1);

}

.cell:hover { transform: scale(0.96); filter: brightness(1.05); }

.cell:active { transform: scale(0.90); }

.color-0 {

background-color: var(--cyan-neon);

box-shadow: 0 2px 8px rgba(0, 229, 201, 0.35);

}

.color-1 {

background-color: var(--blue-neon);

box-shadow: 0 2px 8px rgba(14, 165, 233, 0.35);

}

.color-2 {

background-color: var(--orange-neon);

box-shadow: 0 2px 8px rgba(249, 115, 22, 0.35);

}

.btn-group {

display: flex;

gap: 10px;

max-width: 320px;

margin: 0 auto;

}

.upload-btn {

flex: 2;

background: var(--primary-color);

border: none;

color: #ffffff;

padding: 12px;

border-radius: 30px;

font-size: 12px;

font-weight: 700;

letter-spacing: 0.8px;

text-transform: uppercase;

cursor: pointer;

box-shadow: 0 4px 12px rgba(14, 165, 233, 0.25);

transition: all 0.2s ease;

}

.upload-btn:hover {

background: var(--primary-hover);

transform: translateY(-1px);

box-shadow: 0 6px 16px rgba(14, 165, 233, 0.35);

}

.upload-btn:disabled { opacity: 0.5; cursor: not-allowed; }

.reset-btn {

flex: 1;

background: #f1f5f9;

border: 1px solid #e2e8f0;

color: #475569;

padding: 12px;

border-radius: 30px;

font-size: 12px;

font-weight: 600;

cursor: pointer;

transition: all 0.2s ease;

}

.reset-btn:hover { background: #e2e8f0; color: #0f172a; }

/* Status Card */

.status-card {

margin-top: 18px;

background: var(--panel-bg);

padding: 16px 20px;

border-radius: 14px;

border: 1px solid var(--border-color);

}

.status-card h5 { font-size: 13px; margin-bottom: 4px; font-weight: 700; color: #1e293b; }

.status-card p { font-size: 12px; color: var(--text-muted); margin-bottom: 10px; }

.color-legend {

display: flex;

align-items: center;

gap: 14px;

}

.legend-item { display: flex; align-items: center; gap: 6px; font-size: 11px; color: var(--text-muted); font-weight: 500; }

.color-dot { width: 12px; height: 12px; border-radius: 3px; }

/* Right Column: Key & Decryption */

.access-card {

background: var(--panel-bg);

border: 1px solid var(--border-color);

border-radius: 18px;

padding: 22px;

margin-bottom: 22px;

}

.input-label { display: block; font-size: 12px; color: var(--text-muted); margin-bottom: 8px; font-weight: 600; }

.text-input {

width: 100%;

background: var(--input-bg);

border: 1px solid var(--border-color);

color: #0f172a;

padding: 13px 16px;

border-radius: 10px;

font-size: 14px;

margin-bottom: 14px;

outline: none;

transition: border-color 0.2s, box-shadow 0.2s;

}

.text-input:focus {

border-color: var(--blue-neon);

box-shadow: 0 0 0 3px rgba(14, 165, 233, 0.12);

}

.decrypt-btn {

width: 100%;

background: #0f172a;

color: #ffffff;

border: none;

padding: 13px;

border-radius: 10px;

font-size: 13px;

font-weight: 700;

letter-spacing: 0.8px;

text-transform: uppercase;

cursor: pointer;

box-shadow: 0 4px 12px rgba(15, 23, 42, 0.15);

transition: all 0.2s ease;

}

.decrypt-btn:hover {

background: #1e293b;

transform: translateY(-1px);

box-shadow: 0 6px 16px rgba(15, 23, 42, 0.2);

}

.decrypt-card {

background: var(--panel-bg);

border: 1px solid var(--border-color);

border-radius: 18px;

padding: 22px;

min-height: 155px;

}

.decrypted-title { font-size: 13px; color: var(--text-muted); margin-bottom: 12px; font-weight: 500; }

.decrypted-text {

font-size: 15px;

font-weight: 600;

color: #0369a1;

background: #e0f2fe;

border-left: 3px solid #0284c7;

padding: 14px 16px;

border-radius: 0 8px 8px 0;

line-height: 1.5;

display: none;

animation: fadeIn 0.3s ease-out;

}

@keyframes fadeIn {

from { opacity: 0; transform: translateY(4px); }

to { opacity: 1; transform: translateY(0); }

}

.msg-feedback { font-size: 12px; margin-top: 10px; min-height: 16px; font-weight: 600; }

</style>

</head>

<body>

<div class="app-container">

<div class="header-logo">

<div class="logo-icon">

<div class="dot-c"></div><div class="dot-b"></div><div class="dot-o"></div>

<div class="dot-b"></div><div class="dot-o"></div><div class="dot-c"></div>

<div class="dot-o"></div><div class="dot-c"></div><div class="dot-b"></div>

</div>

<div class="logo-text">

<h4>COLOR CODE</h4>

<p>SECURE</p>

</div>

</div>

<h1 class="main-title">5x5 Pattern Recognition and Data Decryption</h1>

<div class="two-column-layout">

<!-- Left Column: Pattern Recognition -->

<div>

<div class="step-title">Step 1: Pattern Recognition</div>

<!-- Interactive Grid -->

<div class="grid-container" id="gridContainer"></div>

<div class="btn-group">

<!-- Pure album upload button: Removed the "capture" attribute to prevent the phone from automatically launching the direct photo-taking function. -->

<button id="uploadBtn" class="upload-btn" onclick="document.getElementById('photoInput').click()">Upload Pattern Photo</button>

<button class="reset-btn" onclick="resetGrid()">Reset</button>

<input type="file" id="photoInput" accept="image/*" style="display:none" onchange="processImageWithCV(event)">

</div>

<div class="status-card">

<h5 id="statusHeader">PATTERN STATE</h5>

<p id="statusDesc">Click blocks to edit, or <b>upload a clear photo from your gallery</b>.</p>

<div class="color-legend">

<div class="legend-item"><div class="color-dot color-0"></div>Cyan</div>

<div class="legend-item"><div class="color-dot color-1"></div>Blue</div>

<div class="legend-item"><div class="color-dot color-2"></div>Orange</div>

</div>

</div>

</div>

<!-- Right Column: Access & Decryption -->

<div>

<div class="step-title">Step 2: Access & Decryption</div>

<div class="access-card">

<label class="input-label">Security Key</label>

<input type="text" id="secretKeyInput" class="text-input" placeholder="Enter Key...">

<button class="decrypt-btn" onclick="executeDecryption()">Decrypt Data</button>

<div id="decryptFeedback" class="msg-feedback"></div>

</div>

<div class="step-title">Decrypted Information</div>

<div class="decrypt-card">

<div class="decrypted-title">The decrypted information is:</div>

<div id="decryptedOutput" class="decrypted-text">

force-triggered bidirectional tricolor fluorescence switches

</div>

</div>

</div>

</div>

</div>

<script>

const TARGET_PATTERN = [

0, 2, 2, 0, 2,

1, 0, 0, 0, 2,

1, 0, 0, 1, 0,

0, 2, 2, 2, 0,

1, 0, 0, 1, 1

];

let isOpenCvReady = false;

let currentPattern = new Array(25).fill(0);

const gridContainer = document.getElementById('gridContainer');

function onCvReady() {

isOpenCvReady = true;

}

function initGrid() {

gridContainer.innerHTML = '';

for (let i = 0; i < 25; i++) {

const cell = document.createElement('div');

cell.className = `cell color-${currentPattern[i]}`;

cell.title = "Click to toggle color";

cell.onclick = () => {

currentPattern[i] = (currentPattern[i] + 1) % 3;

renderGrid();

validatePattern();

};

gridContainer.appendChild(cell);

}

}

function renderGrid() {

const cells = gridContainer.getElementsByClassName('cell');

for (let i = 0; i < 25; i++) {

cells[i].className = `cell color-${currentPattern[i]}`;

}

}

function resetGrid() {

currentPattern = new Array(25).fill(0);

renderGrid();

validatePattern();

document.getElementById('secretKeyInput').value = '';

document.getElementById('photoInput').value = '';

document.getElementById('decryptedOutput').style.display = 'none';

document.getElementById('decryptFeedback').innerText = '';

}

// The first anchor point calibration algorithm (based on the first block as the standard cyan reference)

function applyReferenceClassification(blockStats) {

const detected = [];

const refCyan = blockStats[0]; // Take the first square in the upper left corner as the standard cyan anchor point

const refRatioGB = refCyan.avgG / (refCyan.avgB + 0.001);

blockStats.forEach(stat => {

// 1. Prioritize the removal of the orange ones

if (stat.avgR > stat.avgB * 1.2 && stat.avgR > 80) {

detected[stat.index] = 2; // Orange

} else {

// 2. Relative white balance comparison: Binary determination of cyan and blue colors

const currentRatioGB = stat.avgG / (stat.avgB + 0.001);

if (currentRatioGB > refRatioGB * 0.75) {

detected[stat.index] = 0; // Cyan

} else {

detected[stat.index] = 1; // Blue

}

}

});

currentPattern = detected;

renderGrid();

validatePattern();

}

function processImageWithCV(event) {

const file = event.target.files[0];

if (!file) return;

const reader = new FileReader();

reader.onload = function(e) {

const img = new Image();

img.onload = function() {

const MAX_SIZE = 800;

let width = img.width;

let height = img.height;

if (width > height && width > MAX_SIZE) {

height *= MAX_SIZE / width;

width = MAX_SIZE;

} else if (height > MAX_SIZE) {

width *= MAX_SIZE / height;

height = MAX_SIZE;

}

const canvas = document.createElement('canvas');

const ctx = canvas.getContext('2d');

canvas.width = width;

canvas.height = height;

ctx.drawImage(img, 0, 0, width, height);

if (!isOpenCvReady || typeof cv === 'undefined') {

fallbackBoundingBox(canvas, ctx);

return;

}

try {

const src = cv.imread(canvas);

const gray = new cv.Mat();

const thresh = new cv.Mat();

const contours = new cv.MatVector();

const hierarchy = new cv.Mat();

cv.cvtColor(src, gray, cv.COLOR_RGBA2GRAY, 0);

cv.threshold(gray, thresh, 0, 255, cv.THRESH_BINARY + cv.THRESH_OTSU);

cv.findContours(thresh, contours, hierarchy, cv.RETR_EXTERNAL, cv.CHAIN_APPROX_SIMPLE);

const candidateBlocks = [];

const minArea = (width * height) * 0.002;

const maxArea = (width * height) * 0.08;

for (let i = 0; i < contours.size(); ++i) {

const rect = cv.boundingRect(contours.get(i));

const area = rect.width * rect.height;

const aspect = rect.width / rect.height;

if (area > minArea && area < maxArea && aspect >= 0.55 && aspect <= 1.8) {

candidateBlocks.push(rect);

}

}

if (candidateBlocks.length >= 20) {

candidateBlocks.sort((a, b) => a.y - b.y);

const rows = [];

const rowCount = 5;

const itemsPerRow = Math.round(candidateBlocks.length / rowCount);

for (let r = 0; r < rowCount; r++) {

const rowSlice = candidateBlocks.slice(r * itemsPerRow, (r + 1) * itemsPerRow);

rowSlice.sort((a, b) => a.x - b.x);

rows.push(...rowSlice.slice(0, 5));

}

const blockStats = [];

rows.slice(0, 25).forEach((rect, idx) => {

const sampleW = Math.max(2, Math.floor(rect.width * 0.4));

const sampleH = Math.max(2, Math.floor(rect.height * 0.4));

const startX = Math.floor(rect.x + (rect.width - sampleW) / 2);

const startY = Math.floor(rect.y + (rect.height - sampleH) / 2);

const pData = ctx.getImageData(startX, startY, sampleW, sampleH).data;

let sR = 0, sG = 0, sB = 0;

const totalP = sampleW * sampleH;

for (let p = 0; p < pData.length; p += 4) {

sR += pData[p];

sG += pData[p + 1];

sB += pData[p + 2];

}

blockStats.push({ index: idx, avgR: sR/totalP, avgG: sG/totalP, avgB: sB/totalP });

});

applyReferenceClassification(blockStats);

src.delete(); gray.delete(); thresh.delete(); contours.delete(); hierarchy.delete();

return;

}

let maxBox = null;

let maxBoxArea = 0;

for (let i = 0; i < contours.size(); ++i) {

const rect = cv.boundingRect(contours.get(i));

const area = rect.width * rect.height;

const aspect = rect.width / rect.height;

if (aspect >= 0.6 && aspect <= 1.5 && area > maxBoxArea) {

maxBoxArea = area;

maxBox = rect;

}

}

if (maxBox) {

sampleFromBoundingBox(ctx, maxBox.x, maxBox.y, maxBox.width, maxBox.height);

} else {

fallbackBoundingBox(canvas, ctx);

}

src.delete(); gray.delete(); thresh.delete(); contours.delete(); hierarchy.delete();

} catch (err) {

fallbackBoundingBox(canvas, ctx);

}

};

img.src = e.target.result;

};

reader.readAsDataURL(file);

}

function sampleFromBoundingBox(ctx, x, y, w, h) {

const padX = w * 0.03;

const padY = h * 0.03;

const cropX = x + padX;

const cropY = y + padY;

const cropW = w - 2 * padX;

const cropH = h - 2 * padY;

const stepX = cropW / 5;

const stepY = cropH / 5;

const blockStats = [];

for (let row = 0; row < 5; row++) {

for (let col = 0; col < 5; col++) {

const startX = Math.floor(cropX + col * stepX + stepX * 0.25);

const startY = Math.floor(cropY + row * stepY + stepY * 0.25);

const sampleW = Math.max(1, Math.floor(stepX * 0.5));

const sampleH = Math.max(1, Math.floor(stepY * 0.5));

const blockData = ctx.getImageData(startX, startY, sampleW, sampleH).data;

let sumR = 0, sumG = 0, sumB = 0, validCount = 0;

for (let p = 0; p < blockData.length; p += 4) {

if (blockData[p] < 20 && blockData[p+1] < 20 && blockData[p+2] < 20) continue;

sumR += blockData[p];

sumG += blockData[p + 1];

sumB += blockData[p + 2];

validCount++;

}

const avgR = validCount > 0 ? sumR / validCount : 0;

const avgG = validCount > 0 ? sumG / validCount : 0;

const avgB = validCount > 0 ? sumB / validCount : 0;

blockStats.push({ index: row * 5 + col, avgR, avgG, avgB });

}

}

applyReferenceClassification(blockStats);

}

function fallbackBoundingBox(canvas, ctx) {

const w = canvas.width, h = canvas.height;

sampleFromBoundingBox(ctx, w * 0.15, h * 0.25, w * 0.75, h * 0.55);

}

function validatePattern() {

const isMatched = currentPattern.every((val, idx) => val === TARGET_PATTERN[idx]);

const header = document.getElementById('statusHeader');

const desc = document.getElementById('statusDesc');

if (isMatched) {

header.innerText = "PHOTO RECOGNIZED:";

header.style.color = "#0ea5e9";

desc.innerText = "Matching Pattern Found. Proceed to Step 2.";

desc.style.color = "#0f172a";

} else {

header.innerText = "PATTERN MISMATCH";

header.style.color = "#ef4444";

desc.innerText = "Click cells to manually adjust or re-upload photo.";

desc.style.color = "var(--text-muted)";

}

return isMatched;

}

function executeDecryption() {

const isMatched = validatePattern();

const key = document.getElementById('secretKeyInput').value.trim();

const feedback = document.getElementById('decryptFeedback');

const output = document.getElementById('decryptedOutput');

if (!isMatched) {

feedback.style.color = "#ef4444";

feedback.innerText = "Pattern recognition must match first.";

output.style.display = "none";

return;

}

if (key === "JSTNU") {

feedback.style.color = "#10b981";

feedback.innerText = "Access granted. Payload decrypted successfully.";

output.style.display = "block";

} else {

feedback.style.color = "#ef4444";

feedback.innerText = "Invalid security key. Access denied.";

output.style.display = "none";

}

}

initGrid();

</script>

</body>

</html>

**Figures and Tables**

**Scheme S1.** Synthetic routes of 20 **R_1_-R_2_** compounds.

**Figure S1.** CIE chromaticity diagrams of luminogen **PhCz_3_-*m*-CF_3_** in different solid states: (a) unground, (b) ground.

**Figure S2.** (a) Normalized PL spectra and PL photographs under 365 nm UV excitation of **PhCz_3_-*p*-CF_3_** before and after grinding. (b) CIE chromaticity diagrams of **PhCz_3_-*p*-CF_3_** before and after grinding.

**Figure S3.** CIE chromaticity diagrams of **PhCz_3_-*o*-CF_3_** before and after grinding.

**Figure S4.** (a) Normalized PL spectra and PL photographs under 365 nm UV excitation of **TPE-H** in various solid states. (b) CIE chromaticity diagrams of **TPE-H** before and after grinding.

**Figure S5.** (a) Normalized PL spectra and PL photographs under 365 nm UV excitation of **TPE-*m*-CF_3_** in various solid states. (b) CIE chromaticity diagrams of **TPE-*m*-CF_3_** before and after grinding.

**Figure S6.** (a) Normalized PL spectra and PL photographs under 365 nm UV excitation of **TPE-*p*-CF_3_** in various solid states. (b) CIE chromaticity diagrams of **TPE-*p*-CF_3_** before and after grinding.

**Figure S7.** (a) Normalized PL spectra and PL photographs under 365 nm UV excitation of **CzPh-H** in various solid states. (b) CIE chromaticity diagrams of **CzPh-H** before and after grinding.

**Figure S8.** CIE chromaticity diagrams of **CzPh-*o*-CF_3_** in various solid states.

**Figure S9.** (a) Normalized PL spectra and PL photographs under 365 nm UV excitation of **Py-*o*-CF_3_** in various solid states. (b) CIE chromaticity diagrams of **Py-*o*-CF_3_** in various solid states.

**Figure S10.** (a) Normalized PL spectra and PL photographs under 365 nm UV excitation of **Py-*m*-CF_3_** in various solid states. (b) CIE chromaticity diagrams of **Py-*m*-CF_3_** in various solid states.

**Figure S11.** (a) Normalized PL spectra and PL photographs under 365 nm UV excitation of **Py-*p*-CF_3_** in various solid states. (b) CIE chromaticity diagrams of **Py-*p*-CF_3_** in various solid states.

**Figure S12.** (a) Normalized PL spectra and PL photographs under 365 nm UV excitation of **CzPh-*m*-CF_3_** in various solid states. (b) CIE chromaticity diagrams of **CzPh-*m*-CF_3_** in various solid states.

**Figure S13.** (a) Normalized PL spectra and PL photographs under 365 nm UV excitation of **CzPh-*p*-CF_3_** in various solid states. (b) CIE chromaticity diagrams of **CzPh-*p*-CF_3_** in various solid states.

**Figure S14.** (a) Normalized PL spectra and PL photographs under 365 nm UV excitation of **PhCz2-H** in various solid states. (b) CIE chromaticity diagrams of **PhCz2-H** in various solid states.

**Figure S15.** (a) Normalized PL spectra and PL photographs under 365 nm UV excitation of **PhCz2-*o*-CF_3_** in various solid states. (b) CIE chromaticity diagrams of **PhCz2-*o*-CF_3_** in various solid states.

**Figure S16.** (a) Normalized PL spectra and PL photographs under 365 nm UV excitation of **PhCz2-*m*-CF_3_** in various solid states. (b) CIE chromaticity diagrams of **PhCz2-*m*-CF_3_** in various solid states.

**Figure S17.** (a) Normalized PL spectra and PL photographs under 365 nm UV excitation of **PhCz3-H** in various solid states. (b) CIE chromaticity diagrams of **PhCz3-H** in various solid states.

**Figure S18.** CIE chromaticity diagrams of **TPE-*o*-CF_3_** in various solid states.

**Figure S19.** (a) Normalized PL spectra and PL photographs under 365 nm UV excitation of **Py-H** in various solid states. (b) CIE chromaticity diagrams of **Py-H** in various solid states.

**Figure S20.** (a) Normalized PL spectra and PL photographs under 365 nm UV excitation of **PhCz2-*p*-CF_3_** in various solid states. (b) CIE chromaticity diagrams of **PhCz2-*p*-CF_3_** in various solid states.

**Figure S21.** The dihedral angles between the central dibenzofulvene core and the trifluoromethylbenzene units of (a) **Py-*o*-CF_3_** crystal and (b) **TPE-*o*-CF_3_** crystal.

**Figure S22.** 2D fingerprint plots of compound **Py-*o*-CF_3_**: full (a), and resolved into C∙∙∙H (b) and F∙∙∙H (c) contacts showing the percentages of contacts contributed to the total Hirshfeld surface area of molecule. 2D fingerprint plots of compound **TPE-*o*-CF_3_**: full (d), and resolved into C∙∙∙H (e) and F∙∙∙H (f) contacts showing the percentages of contacts contributed to the total Hirshfeld surface area of molecule. The grey shadow is an outline of the complete fingerprint plot for each plot.

**Figure S23.** Optimized geometry of the **TPE-*o*-CF_3_** crystal in its dimeric form calculated at the B3LYP-D3BJ [60-63]/6-31G* level of theory.

**Figure S24.** Demonstration of the decryption platform recognizing the 5×5 fluorescence matrix before grinding.

**Figure S25.** Demonstration of the decryption platform recognizing the 5×5 fluorescence matrix after slight grinding.

**Figure S26.** Demonstration of the decryption platform recognizing the 5×5 fluorescence matrix after heavy grinding.

**Tabel S1.** The *λ*_max_ values, absolute fluorescence quantum yields (*Φ*_F_) and fluorescence lifetimes of 20 new **R_1_-R_2_** luminogens in different solid states.

**Table S2**. Crystal data of **Py-*o*-CF_3_** and **TPE-*o*-CF_3_**.

**Table S3**. Multiple intermolecular interactions summarizations of stacking structures of **Py-*o*-CF_3_** crystal and **TPE-*o*-CF_3_** crystal.

**Table S4**. Major eclectronic excitations for **TPE-*o*-CF_3_** crystal in its dimeric form.

Copies of NMR spectra and HRMS spectra of compounds R_1_-R_2_

^1^H NMR spectrum of **Py-H**

^13^C NMR spectrum of **Py-H**

HRMS spectrum of **Py-H**

^1^H NMR spectrum of **Py-*o*-CF_3_**

^13^C NMR spectrum of **Py-*o*-CF_3_**

HRMS spectrum of **Py-*o*-CF_3_**

^1^H NMR spectrum of **Py-*m*-CF_3_**

^13^C NMR spectrum of **Py-*m*-CF_3_**

HRMS spectrum of **Py-*m*-CF_3_**

^1^H NMR spectrum of **Py-*p*-CF_3_**

^13^C NMR spectrum of **Py-*p*-CF_3_**

HRMS spectrum of **Py-*p*-CF_3_**

^1^H NMR spectrum of **TPE-H**

^13^C NMR spectrum of **TPE-H**

HRMS spectrum of **TPE-H**

^1^H NMR spectrum of **TPE-*o*-CF_3_**

^13^C NMR spectrum of **TPE-*o*-CF_3_**

HRMS spectrum of **TPE-*o*-CF_3_**

^1^H NMR spectrum of **TPE-*m*-CF_3_**

^13^C NMR spectrum of **TPE-*m*-CF_3_**

HRMS spectrum of **TPE-*m*-CF_3_**

^1^H NMR spectrum of **TPE-*p*-CF_3_**

^13^C NMR spectrum of **TPE-*p*-CF_3_**

HRMS spectrum of **TPE-*p*-CF_3_**

^1^H NMR spectrum of **CzPh-H**

^13^C NMR spectrum of **CzPh-H**

HRMS spectrum of **CzPh-H**

^1^H NMR spectrum of **CzPh-*o*-CF_3_**

^13^C NMR spectrum of **CzPh-*o*-CF_3_**

HRMS spectrum of **CzPh-*o*-CF_3_**

^1^H NMR spectrum of **CzPh-*m*-CF_3_**

^13^C NMR spectrum of **CzPh-*m*-CF_3_**

HRMS spectrum of **CzPh-*m*-CF_3_**

^1^H NMR spectrum of **CzPh-*p*-CF_3_**

^13^C NMR spectrum of **CzPh-*p*-CF_3_**

HRMS spectrum of **CzPh-*p*-CF_3_**

^1^H NMR spectrum of **PhCz2-H**

^13^C NMR spectrum of **PhCz2-H**

HRMS spectrum of **PhCz2-H**

^1^H NMR spectrum of **PhCz2-*o*-CF_3_**

^13^C NMR spectrum of **PhCz2-*o*-CF_3_**

HRMS spectrum of **PhCz2-*o*-CF_3_**

^1^H NMR spectrum of **PhCz2-*m*-CF_3_**

^13^C NMR spectrum of **PhCz2-*m*-CF_3_**

HRMS spectrum of **PhCz2-*m*-CF_3_**

^1^H NMR spectrum of **PhCz2-*p*-CF_3_**

^13^C NMR spectrum of **PhCz2-*p*-CF_3_**

HRMS spectrum of **PhCz2-*p-*CF_3_**

^1^H NMR spectrum of **PhCz3-H**

^13^C NMR spectrum of **PhCz3-H**

HRMS spectrum of **PhCz3-H**

^1^H NMR spectrum of **PhCz3-*o*-CF_3_**

^13^C NMR spectrum of **PhCz3-*o*-CF_3_**

HRMS spectrum of **PhCz3-*o*-CF_3_**

^1^H NMR spectrum of **PhCz3-*m*-CF_3_**

^13^C NMR spectrum of **PhCz3-*m*-CF_3_**

HRMS spectrum of **PhCz3-*m*-CF_3_**

^1^H NMR spectrum of **PhCz3-*p*-CF_3_**

^13^C NMR spectrum of **PhCz3-*p*-CF_3_**

HRMS spectrum of **PhCz3-*p*-CF_3_**

# References

[1] J. Xu, S. Semin, D. Niedzialek, at al., “Self-Assembled Organic Microfibers for Nonlinear Optics,” *Advanced Materials* 25 no. 14 (2013): 2084-2089, https://doi.org/10.1002/adma.201204237.
[2] L. Zong, J. Wang, X. Jian, C. Liu, N. Li, and L. Zhu, “Fluorenyl-Containing Bisphenol Monomer and Fluorenyl Polyarylether Resin as well as Preparation Method and Application Thereof,” CN 117209367 A (2023).
[3] S. Li, X. Kang, D. Xu, at al., “Novel AIE-Active Dicyano-Bridged Dinuclear Gold(I) Complexes: Bathochromic Mechanofluorochromism, Mechanofluorochromic Quenching and Its Application in Anticounterfeit,” *Journal of Molecular Structure* 1373 (2026): 146709, https://doi.org/[10.1016/j.molstruc.2026.146709](https://doi.org/10.1016/j.molstruc.2026.146709).
[4] S. Tan, Y. Yin, W. Chen, Z. Chen, W. Tian, and S. Pu, “Carbazole-Based Highly Solid-State Emissive Fluorene Derivatives with Various Mechanochromic Fluorescence Characteristics,” [*Dyes and Pigments*](https://www.sciencedirect.com/journal/dyes-and-pigments) 177 (2020): 108302, https://doi.org/[10.1016/j.dyepig.2020.108302](https://doi.org/10.1016/j.dyepig.2020.108302).
